# Hyaluronic acid as a versatile building block for the development of biofunctional hydrogels: In vitro models and preclinical innovations

**DOI:** 10.1016/j.mtbio.2025.101596

**Published:** 2025-02-18

**Authors:** Noémie Petit, Yu-yin Joanne Chang, Franz Acker Lobianco, Tom Hodgkinson, Shane Browne

**Affiliations:** a Tissue Engineering Research Group, Department of Anatomy and Regenerative Medicine, Royal College of Surgeons in Ireland, 123, St Stephen's Green, Dublin 2, Ireland; b CÚRAM, Research Ireland Centre for Medical Devices, University of Galway, Galway, H91 W2TY, Ireland; c Trinity Centre for Biomedical Engineering, Trinity College Dublin, Dublin 2, Ireland

**Keywords:** Hyaluronic acid, Hydrogels, Biomaterial modification and functionalisation, In vitro models, Therapeutic interventions

## Abstract

Hyaluronic acid (HyA) is a non-sulphated linear polysaccharide found abundantly in the extracellular matrix, known for its biocompatibility and versatility in tissue engineering. Chemical modifications of HyA, including methacrylate, acrylate, click chemistry, norbornene, or host-guest chemistry, are necessary for the formation of stable hydrogels with tuneable biophysical characteristics. These modifications enable precise control over stiffness, swelling, degradation, and advanced functionalities such as shear-thinning, self-healing, and injectability. Functionalisation further enhances hydrogel bioactivity, enabling controlled cell adhesion, modulation of cell behaviour, hydrogel degradation, and release profiles, as well as inflammation modulation or bacterial growth inhibition. These are achieved by conjugating proteins, peptides, antibodies, or reactive chemical groups. HyA hydrogels find broad applications both *in vitro* and *in vivo*. *In vitro*, HyA-based hydrogels can support the development of models to understand fundamental processes in health and mechanisms behind disease progression, serving as highly tuneable extracellular matrix mimetics. As therapeutic interventions, injectable or implantable HyA-based hydrogels have been developed to repair a range of tissues, including cartilage, bone, muscle, and skin defects. However, issues remain to be addressed before widespread adoption of HyA-based hydrogels as clinical options. Future innovations for HyA hydrogels include its establishment as an enabling technology for the delivery of novel therapeutics, with a particular focus on immunomodulatory molecules, and the development of more dynamic, tissue-mimetic HyA-based hydrogels.

## Highlights

• Hyaluronic acid (HyA)'s biocompatibility and tuneability enable diverse applications.
• A range of modifications facilitate formation of hydrogels with tuneable properties.
• HyA hydrogels can be functionalised with biomolecules for added bioactivity.
• HyA hydrogels are widely used *in vitro* for modelling healthy and diseased tissue.
• HyA hydrogels are used as implantable or injectable biomaterials for tissue repair.

## Graphical abstract

Created in BioRender. Chang, J. (2025) https://BioRender.com/z32q422.

**Figure:**
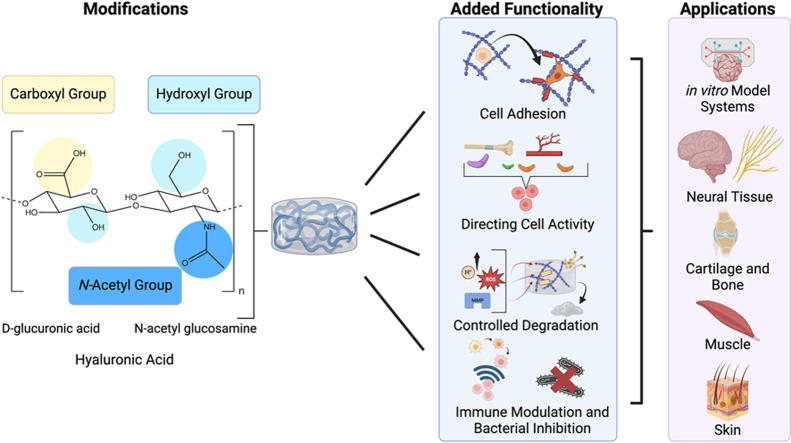
Hyaluronic acid is a versatile building block for the development of biofunctional hydrogels. Chemical modifications of hyaluronic acid enable tunable biophysical and biochemical properties, facilitating diverse functionalities such as cell adhesion, controlled degradation, and immune modulation. These engineered hydrogels support applications in in vitro models, neural tissue engineering, cartilage and bone regeneration, muscle repair, and skin tissue engineering.

## Abbreviations

**AcHyA**: acrylate-modified hyaluronic acid
**ACuBG**: copper-doped bioglass
**Ad**: adamantane
**ADH**: adipic acid dihydrazide
**Ad-HA**: adamantane-modified HyA
**AFnSi**: acrylate-functionalised nano-silica
**AHA**: aminated hyaluronic acid
**AMP**: antimicrobial peptide
**β-CD**: β-cyclodextrin
**β-TCP**: β-tricalcium phosphate
**BDNF**: brain-derived neurotrophic factor
**bFGF**: basic fibroblast growth factor
**BMP-2**: bone morphogenetic protein 2
**BMSC**: bone mesenchymal stem cells
**Boc2O**: di-*tert*-butyl dicarbonate
**BOP**: (Benzotriazol-1-yloxy)tris(dimethylamino)phosphonium hexafluorophosphate
**CD-HA**: β-CD modified hyaluronic acid
**CNS**: central nervous system
**CPC**: cardiac progenitor cells
**CSPG**: chondroitin sulphate proteoglycan
**DBCO**: dibenzocyclooctyne
**DMAP**: dimethylaminopyridine
**DMF**: dimethylformamide
**DMSO**: dimethyl sulphoxide
**DMTMM**: 4-(4,6-dimethoxy-1,3,5-triazin-2-yl)-4-methylmorpholinium chloride
**DOX**: doxorubicin
**DS**: degree of substitution
**DTT**: dithiothreitol
**DVS**: divinylsulphone
**ECFC**: endothelial colony-forming cells
**ECM**: extracellular matrix
**EDC**: *N*-(3-dimethylaminopropyl)-*N′*-ethylcarbodiimide
**EGCG**: epigallocatechin-3-gallate
**EGF**: epidermal growth factor
**EGFR**: epidermal growth factor receptor
**EPL**: ε-Polylysine
**EV**: extracellular vesicle
**FCSA**: fibre cross-sectional area
**GAG**: glycosaminoglycan
**GelMA**: gelatin-methacrylate
**Gel-SH**: thiolated gelatin
**GFAP**: glial fibrillary acidic protein
**H**_**2**_**O**_**2**_: hydrogen peroxide
**HA-ADH**: hydrazide-modified hyaluronic acid
**HAD**: human amnion-derived
**HA-DVS**: divinyl sulfone-modified hyaluronic acid
**HA-CHO**: aldehyde-modified hyaluronic acid
**HA-DBCO**: dibenzocyclooctyne-modified hyaluronic acid
**HA-dCHO**: dialdehyde-modified hyaluronic acid
**hADSC**: human adipose-derived stem cells
**HA-mCHO**: monoaldehyde-modified hyaluronic acid
**HA-SH**: thiol-modified hyaluronic acid
**HA-TBA**: tetrabutylammonium salt of hyaluronic acid
**HA-Tyr**: tyramine-modified hyaluronic acid
**HA-VS**: vinyl sulphonated hyaluronic acid
**HDF**: human dermal fibroblasts
**HEA**: 2-hydroxyethyl acrylate
**HEPES**: 4-(2-hydroxyethyl)-1-piperazine ethanesulphonic acid
**hESC**: human embryonic stem cells
**hiPSC**: human induced pluripotent stem cells
**hMSC**: human mesenchymal stem cells
**HOBt**: *N*-hydroxybenzotriazole
**hPDLC**: human periodontal ligament cells
**HRP**: horseradish peroxidase
**HUVEC**: human umbilical vein endothelial cells
**HyA**: hyaluronic acid
**HyAp**: hydroxyapatite
**LAP**: lithium phenyl-2,4,6-trimethylbenzoylphosphinate
**MA**: maleic anhydride
**MAHA**: maleate-modified hyaluronic acid
**MAPK**: mitogen-activated protein kinase
**MBP**: myelin basic protein
**MeHA**: methacrylate-modified hyaluronic acid
**MES**: 2-(*N*-morpholino)-ethanesulphonic acid
**MLT**: melatonin
**MMP**: matrix metalloproteinase
**MW**: molecular weight
**NAS**: *N*-acryloxysuccinimide
**NGF**: nerve growth factor
**NHS**: *N*-hydroxysuccinimide
**NorHA**: norbornene-modified hyaluronic acid
**NPC**: neural progenitor cells
**NSC**: neural stem cells
**OPC**: oligodendrocyte progenitor cells
**PANI**: polyaniline
**PCL**: polycaprolactone
**PDA**: polydopamine
**PDGF**: platelet-derived growth factor
**PEG**: poly(ethylene glycol)
**PEG-SH_4_**: PEG tetra-thiols
**PEGDA**: PEG diacrylate
**PGA**: polyglutamic acid
**PLGA**: poly(lactic-co-glycolic acid)
**PLL**: poly-L-lysine
**PRP**: platelet-rich plasma
**RGD**: arginylglycylaspartic acid
**rhAm**: recombinant human amelogenin
**ROS**: reactive oxygen species
**SO**_**3**_: sulphur trioxide
**SPAAC**: strain-promoted azide-alkyne cycloaddition
**TA**: tibialis anterior
**TBA**: tetrabutylammonium
**TEMED**: *N*,*N*,*N*′,*N*′-tetramethylethylenediamine
**VEGF**: vascular endothelial growth factor
**VML**: volumetric muscle loss
**VPM**: GCNSVPMSMRGGSNCG

## 1 Introduction

Hydrogels are three-dimensional hydrophilic polymeric networks with a high water content that closely resemble the properties and structure of biological tissues [1,2], leading to their wide applicability in fields including tissue engineering [1,3], drug delivery [4], and the development of 3D *in vitro* models [5,6]. Due to their unique characteristics and the ability to tune their biophysical and biochemical characteristics as appropriate, hydrogels have become a material of choice for many applications in regenerative medicine [[7], [8], [9]].

Hydrogels may be derived from a range of polymeric materials, broadly divided into synthetic polymers and natural biopolymers [10]. Synthetic polymers, while versatile, often face drawbacks such as limited biocompatibility and potential toxicity. In contrast, natural biopolymers like fibrin, collagen, and hyaluronic acid (HyA) are widely favoured due to their inherent biocompatibility and biological relevance [[11], [12], [13], [14], [15]]. Amongst natural biopolymers, HyA in particular stands out for its suitability. HyA is a major component of the extracellular matrix (ECM) of various human tissues, is biocompatible, and bioactive. The bioactivity of HyA is dependent on its molecular weight (MW): high MW HyA (>500 kDa) exhibits immunosuppressive and anti-inflammatory properties, while low MW HyA (<500 kDa) can promote pro-inflammatory phenotypes [16]. Furthermore, unlike many biopolymers, HyA can be easily modified without compromising its structure, allowing precise control over hydrogel biophysical properties to influence cell behaviour and tissue response [17,18].

HyA hydrogels are highly tuneable materials, with properties such as gelation time, stiffness, and mesh size directly influenced by the MW of HyA [7,19]. Hydrogels of higher MW HyA tend to gel faster, are stiffer, and have a smaller mesh size, while lower MW hydrogels demonstrate slower gelation, are softer, and form larger mesh sizes. These variations directly impact the behaviour of encapsulated cells [17]. To further guide cellular behaviour and promote effective tissue regeneration, HyA hydrogels can be customised with bioactive agents [20,21].

To form stable hydrogels, HyA typically undergoes chemical modification to facilitate crosslinking, which is essential to form robust, mechanically-stable hydrogels [7,19]. This process also allows for the conjugation of bioactive molecules, such as peptides, ECM components, and growth factors, which can further enhance the bioactivity of HyA-based hydrogels [20,[22], [23], [24], [25]]. These functionalised HyA-based hydrogels are increasingly being developed for a diverse range of regenerative medicine applications, including wound repair, bone regeneration, limb ischemia, stroke, and corneal repair [24,[26], [27], [28], [29]], due to their inherent safety and low toxicity [30,31].

This review will first discuss the chemical modifications of HyA that facilitate effective hydrogel formation and conjugation of bioactive moieties. It will then discuss recent advancements in functionalisation strategies to enhance bioactivity of HyA hydrogels and direct cell behaviour and tissue repair. Next, it will explore the diverse range of *in vitro* and *in vivo* applications of HyA hydrogels, and conclude with current challenges and future opportunities in HyA-based biomaterials.

## 2 Hyaluronic acid: properties, functions, and applications in hydrogel-based biomaterials

HyA is a naturally occurring, non-sulphated linear polysaccharide found in the native ECM. HyA consists of repeating disaccharide units of *D*-glucuronic acid and *N*-acetyl-*D*-glucosamine linked by β-1-3 and β-1-4 glycosidic bonds [7,20,26]. HyA provides structural support and stability to tissues including the ECM of skin, brain, and cartilage, preserving their shape and elasticity [[32], [33], [34]]. HyA also plays a critical role in maintaining tissue hydration, helping to lubricate joints and maintain the elasticity of the skin and connective tissues [35,36]. Beyond its structural role, HyA also exhibits significant bioactivity, influencing various physiological processes. HyA interacts with cell surface receptors, such as CD44 and receptors for hyaluronic acid-mediated motility, to regulate cellular processes including cell proliferation, migration, and inflammation [37,38]. HyA exists over a wide range of MWs, from less than 6 × 10^3^ Da to more than 1 × 10^6^ Da which impacts its physiological relevance and biodegradability [19]. High levels of HyA in the ECM of foetal tissue have been associated with enhanced tissue healing and scar-free wound repair, further highlighting its potential in regenerative medicine and tissue engineering [[39], [40], [41], [42]].

Clinically, unmodified HyA has been widely used as a filler material in soft tissue replacement and augmentation, particularly in the face and lips [43]. It is also used in surgical procedures for wound healing and in ophthalmic surgeries as a viscoelastic agent [43,44]. In diagnostics, HyA is applied as a contrast agent in ultrasound imaging and in magnetic resonance imaging (MRI), enhancing tissue contrast due to its high water-binding capacity and ability to influence hydration, receptor interactions, and influencing matrix stiffness [45,46]. Together, these clinical applications illustrate the excellent clinical safety profile, versatility, and utility of HyA, which can be further expanded through chemical modifications. The next section will discuss the wide range of chemical modifications applied to HyA to facilitate the formation of hydrogels and support biofunctionalisation strategies.

## 3 Chemical modifications of hyaluronic acid

Unlike other natural biopolymers such as collagen, HyA will not self-assemble into a stable hydrogel. In addition, native HyA is susceptible to rapid enzymatic degradation, resulting in a short half-life following administration [47]. To overcome these limitations, chemical modification of HyA is necessary. These modifications enable the formation of stable HyA-based hydrogels, while bioactive moieties including bioactive peptides, ECM ligands, and other functional groups, can be conjugated to enhance specific bioactivity.

**Fig. 1:**
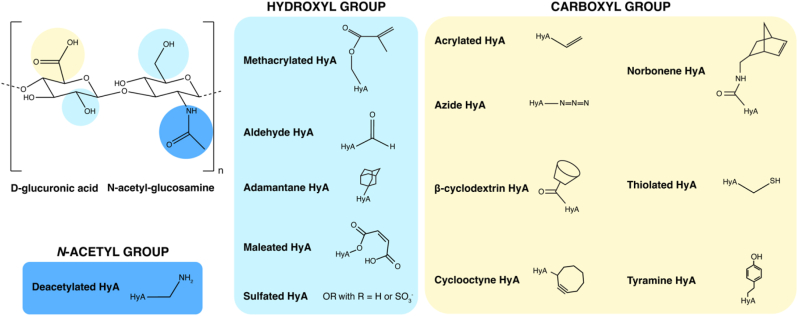
Chemical structure of hyaluronic acid (HyA), chemical groups for modification, and resulting HyA hydrogel precursors.

Several chemical strategies are employed to modify HyA and introduce pendant groups for crosslinking and/or biofunctionalisation. HyA undergoes modification through single bond formation on the three main functional groups: hydroxyl group (-OH), carboxyl group (-COOH), and *N*-acetyl group (-NHCOCH_3_) (Fig. 1). This section will describe a range of chemical modifications of HyA routinely undertaken for biomedical applications.

### 3.1 (Meth)acrylate-modified hyaluronic acid

Methacrylate-modified hyaluronic acid (MeHA) and acrylate-modified hyaluronic acid (AcHyA) are commonly used in biomedical applications to support *in situ* gel formation and functionalisation with bioactive moieties [7]. The ability of MeHA and AcHyA to crosslink upon exposure to light, typically UV or visible light, in the presence of a photoinitiator makes them particularly beneficial in applications such as 3D printing, where photocrosslinking induces hydrogel gelation after deposition [[48], [49], [50]]. This feature allows fabrication of hydrogels with customised stiffness, supporting cell encapsulation, growth, and differentiation.

MeHA is typically synthesised in a straightforward manner by esterification of the hydroxyl group of the *N*-acetyl-*D*-glucosamine unit in HyA methacrylic anhydride at pH 8–9, introducing methacrylate groups (Fig. 1) [[51], [52], [53]]. Addition of a methacrylate group allows for a number of crosslinking strategies, enabling precise control over hydrogel stiffness. For example, hydrogels are formed by UV crosslinking in the presence of a photoinitiator, such as Irgacure 2959, ammonium persulphate, or *N*,*N*,*N*′,*N*′-tetramethylethylenediamine (TEMED) [53]. Reaction of MeHA with dithiothreitol (DTT) initiates a Michael-type addition to form a hydrogel, with the capacity to further adjust hydrogel stiffness with photocrosslinking [54]. MeHA can also be crosslinked without the need for photopolymerization using potassium peroxodisulphate as an initiator and TEMED as catalyst [53]. However, the use of catalysts like TEMED introduces free radicals during polymerisation and causes toxicity [55,56]. Thus, photopolymerization remains the preferred option to crosslink MeHA hydrogels.

AcHyA is typically produced by condensing the carboxyl groups to form an amide bond (Fig. 1). Different strategies have been reported on the formation of the amide linkage onto the backbone of HyA with the most common reaction involving the sequential conjugation of adipic acid dihydrazide (ADH) and *N*-acryloxysuccinimide (NAS) to introduce acrylate groups to the HyA backbone (HA-ADH) [[57], [58], [59]]. These methods typically employ carbodiimide *N*-(3-dimethylaminopropyl)-*N′*-ethylcarbodiimide (EDC) as the carboxyl group-activating condensing agent and NAS as the coupling agent. Segura and co-workers developed a protocol with ADH and EDC under acidic conditions (pH 4.75). After forming the amide bond and the HA-ADH-intermediate, NAS was introduced to the HA-ADH solution in 4-(2-hydroxyethyl)-1-piperazine ethanesulphonic acid (HEPES) buffer (pH 7.2–7.4) to introduce acrylate groups achieving 10–16 % acrylation [60,61]. Adding *N*-hydroxybenzotriazole (HOBt) as an activator with EDC at near-neutral pH increases acrylation [58,62], often exceeding 20 %, by forming active ester intermediates that prevent the formation of unwanted byproducts, facilitating amide bond formation [57,59,63].

Like MeHA, acrylate groups in AcHyA readily react with thiol functional groups through thiol Michael addition or *via* thiol-ene free radical addition (thiol-ene click reactions), and can also undergo photocrosslinking. Thiol-ene chemistry is often used in the formulation of AcHyA-based hydrogels due to the mild reaction conditions and the rapid reactivity of the acrylate groups, particularly through Michael-type addition [64,65]. Hydrogels have been made using thiol-functionalised crosslinkers, including synthetic crosslinker such as poly(ethylene glycol) (PEG)-dithiol, PEG tetra-thiols (PEG-SH_4_), and bis-cysteine-terminated peptides,which can also introduce cell-mediated degradation by incorporating matrix metalloproteinase (MMP)-sensitive sequences [58].

Another shorter, 2-step strategy was employed to synthesise AcHyA. First, 2-hydroxyethyl acrylate (HEA) and succinic anhydride were reacted to obtain HEA-succinate. Concurrently, the tetrabutylammonium salt of HyA (HA-TBA) was generated to enable solubility in organic solvents such as dimethyl sulphoxide (DMSO). Subsequently, HA-TBA was reacted with HEA-succinate in the presence of dimethylaminopyridine (DMAP) and di-*tert*-butyl dicarbonate (Boc2O) to introduce acrylate groups, achieving a degree of acrylation of 38 % [[66], [67], [68]].

A swifter process to modify HyA with an acrylate group has been developed using glycidyl acrylate as the intermediate, yielding a degree of modification of 25 % [69]. This one-step method could prove to be a very efficient and cost-effective approach.

AcHyA and MeHA were sequentially crosslinked *via* Michael-type addition using an MMP-cleavable peptide, followed by photoinitiated radical polymerisation using Irgacure 2959 photoinitiator. This approach allowed encapsulation of human mesenchymal stem cells (hMSCs) in the presence of an adhesive peptide, enabling applications that require spatial control over cell behaviour [67]. Sequential crosslinking could selectively increase hydrogel stiffness to control hMSC spreading within specific regions of the hydrogel.

MeHA and AcHyA have been extensively applied in tissue engineering, drug delivery, and neural tissue engineering [52,70,71].

### 3.2 Aldehyde-modified hyaluronic acid

Aldehyde groups are highly valuable for the formation of stable, *in situ* hydrogels under physiological conditions. Their primary utility lies in their ability to react with amine-containing compounds through a Schiff base reaction, forming imine bonds that enable efficient crosslinking of aldehyde-modified HyA (HA-CHO) hydrogels under mild conditions [47,72].

HA-CHO is typically formed from the oxidation of hydroxyl groups within the glucuronic acid units of HyA, using sodium periodate (Fig. 1) [[73], [74], [75]]. Modifications can be introduced to the HyA backbone to enhance its stability. For example, converting the carboxyl acid groups to diols before oxidation prevents the breakdown of HyA into lower MW fragments. This approach is beneficial because it reduces sugar ring-opening, which can occur during oxidation and lead to degradation [76]. Hozumi et al. demonstrated this by generating HyA-diol intermediates with 3-amino-1,2-propanediol, followed by oxidation to create monoaldehyde-modified-HyA (HA-mCHO), with a modification degree of 12 %. HA-mCHO was crosslinked with carbohydrazide-modified gelatin through Schiff base reaction to form hydrogels, which exhibited greater resistance to degradation than dialdehyde-modified HyA (HA-dCHO), with a modification degree of 4 %, likely due to the ring-opening structure of HA-dCHO being more prone to hydrolysis [77]. As a comparison, HA-mCHO-based hydrogels remained stable for over 3 weeks, in contrast to HA-dCHO-based hydrogels which degraded within 1–2 weeks, highlighting the improved resistance to degradation conferred by the HA-mCHO modification.

In addition to this approach, alternative methods for introducing aldehyde groups have been developed. For instance, carboxyl group can react with L-tartaric acid dihydrazide, followed by periodate oxidation to generate non-enolisable aldehydes with pyruvic-type functionalities. These aldehydes enhance crosslinking stability by increasing intermolecular bonding rather than intramolecular looping. By incorporating both enolisable and non-enolisable aldehydes, the proportion of effective crosslinks can be optimised, which in turn enhances mechanical strength and slows enzymatic degradation by reducing intramolecular looping [78].

HA-CHO hydrogels are typically formed through crosslinking reactions that leverage either amine or thiol chemistry. For instance, thiol-aldehyde reactions can be employed to create hydrogels *via* thiol groups interacting with aldehydes. One example involves crosslinking HA-CHO with polypeptide thiol-modified polyglutamic acid (γ-PGA-SH) to form a dynamic hydrogel. This system uses a cysteine-thiol reaction to form disulphide bonds, while the aldehyde-amine reaction from HA-CHO produces imine bonds. The incorporation of cysteine into this system scavenges reactive oxygen species (ROS), further enhancing the hydrogel's potential for wound healing [79].

Beyond the traditional Schiff base approach, other chemistries can be employed for crosslinking HA-CHO. For instance, thiazolidine chemistry, which involves crosslinking HA-CHO with cysteine-capped ethylenediamine, provides a “reversible-irreversible” mechanism [80]. This two-step reaction first generates an intermediate with shear-thinning and injectable properties, and after injection, the reaction proceeds to form a more stable cyclic thiazolidine structure. This approach not only enhances hydrogel stability but also protects encapsulated cells [80]. The adaptable crosslinking and controlled degradation under physiological conditions of HA-CHO-based hydrogels has led their use as reservoir systems for controlled drug delivery, in cartilage repair, and bioprinting applications [63,[81], [82], [83]].

### 3.3 Cyclooctyne- and azide-modified hyaluronic acid

Click chemistry has gained significant interest for hydrogel development due to high selectivity, yield, and reliability, all essential in biomedical applications [84]. Among the click chemistry reactions, copper-catalysed azide-alkyne cycloaddition has been widely used. However, concerns about the toxicity of copper ions have driven advancements towards copper-free click chemistry. A notable development in this area is the strain-promoted azide-alkyne cycloaddition (SPAAC). SPAAC uses strained cyclooctyne derivatives that react with azides to form triazole linkages without the need for a copper catalyst [85]. This method is advantageous as it is biorthogonal and proceeds efficiently under physiological conditions.

HyA is typically modified with either azide or cyclooctyne groups for hydrogel formation *via* SPAAC. Cyclooctyne-modified HyA is synthesised by modifying the carboxyl groups in HyA with 2-(aminoethoxy)cyclooctyne through amide bond formation, in 2-(*N*-morpholino)-ethanesulphonic acid (MES) buffer, often using 4-(4,6-dimethoxy-1,3,5-triazin-2-yl)-4-methylmorpholinium chloride (DMTMM) as a coupling agent due to its superior activity in aqueous conditions [86,87]. The degree of modification for cyclooctyne-functionalised HyA ranges from 7 % to 25 % [86,87].

On the other hand, azide-modified HyA is created by reacting the carboxyl groups in HyA with azide-bearing reagents such as 11-azidoundecanoic acid or azidopropylamine. These azide groups then participate in click reactions with strained alkyne groups, such as dibenzocyclooctyne (DBCO) or bicyclononyne, which are introduced through carbodiimide chemistry, e.g. EDC-*N*-hydroxysuccinimide (NHS) in MES buffer, or other coupling methods [[87], [88], [89]]. Alternatively, HyA can be modified with DBCO through a two-step bioconjugation process: first, converting HyA to its TBA salt for solubilisation, and then reacting it with DBCO-PEG_4_-NH_2_ using (benzotriazol-1-yloxy)tris(dimethylamino)phosphonium hexafluorophosphate (BOP) as the coupling agent in DMSO, resulting in HA-DBCO [90]. The versatility of SPAAC enables precise functionalisation of HyA, allowing the creation of hydrogels with tailored mechanical properties and degradation rates [91]. This copper-free click chemistry method is particularly beneficial for functionalising HyA-based hydrogels in tissue engineering, immunomodulation, and drug delivery applications [92].

### 3.4 Host-guest chemistry modified hyaluronic acid

Host-guest complexes, a specific form of supramolecular assemblies, are formed when smaller structures are spatially accommodated inside larger molecules [93]. Due to the reversible nature of this type of bond, the resulting hydrogel has fast stress-relaxation, shear-thinning, and self-healing properties. These characteristics are particularly advantageous for 3D bioprinting applications, injectable therapies for tissue regeneration, and drug delivery [94,95].

β-cyclodextrin (β-CD) remains the most popular host molecule for HyA modification. There are three main routes of synthesis to obtain β-CD modified HyA (CD-HA). The first uses diethyl azodicarboxylate and triphenylphosphine as reagents in dioxane using a Mitsunobu reaction [96]. This method uses triphenylphosphine as main reactor molecule, attacking both the carboxylic acid in the HyA chain and a hydroxyl of the β-CD, attaching the β-CD to the carboxylate anion (Fig. 1). This route results in an CD-HA derivative with a 9.2 % reduction in free carboxyl groups compared to native HA [96].

The second route uses EDC∙HCl and NHS in water [97]. The EDC-NHS combination facilitates the reaction of the carboxylic acid group in HyA with an amine form (such as ethylenediamine) of β-CD at pH 5, creating an amide bond to the HyA backbone. The degree of substitution (DS) of β-CD to HA was found to be 15.5 %. This route does not need organic solvents, and different lengths of amines can be used, allowing for more flexible crosslinks in the hydrogel. However, the controlled pH and long reaction times are disadvantageous. A variation of this synthesis that does not require pH control uses DMTMM in MES buffer [98]. Finally, a third route involving BOP produces CD-HA from HA-TBA [95]. TBA is substituted by the BOP molecule, which in turn is susceptible to attack from an amine form of β-CD, leading to amide coupling to HyA. Although this reaction uses DMSO as a solvent, it is not pH-sensitive and takes less than 5 h.

The guest molecule most commonly used with β-CD is adamantane (Ad). The molecular size and structure of Ad makes it a particularly good fit for the hydrophobic inner section of β-CD (Fig. 1). The synthesis of Ad-modified HyA (Ad-HA) uses a two-step reaction where HyA is initially functionalised with TBA [95]. Adding DMAP, Boc2O and 1-adamantaneacetic acid to HA-TBA in DMSO, the reaction follows by the interaction of DMAP and Boc2O generating *tert*-butyl carbonate as by-product. This molecule attacks the TBA, regenerating the carboxyl group, while at the same time DMAP acts as a catalyst for the attachment of Ad to the hydroxyl group on HyA. The modification of HyA repeat units in Ad-HA reaches 32.5 % [95].

Host-guest assemblies allow for the development of HyA-based hydrogels with properties such as shear-thinning, self-healing, and fast stress-relaxation are generated, which are not normally observed in covalently crosslinked materials. For example, supramolecular hydrogels formed from Ad-HA and β-CD exhibit fast stress-relaxation, effectively protecting encapsulated MSCs during mechanical compression [99]. Furthermore, the specificity of the host-guest interactions enables the encapsulation of bioactive molecules, such as peptides, nucleic acids, and growth factors, facilitating gene delivery and tissue regeneration, such as cartilage repair [[99], [100], [101], [102]].

### 3.5 Maleate-modified hyaluronic acid

Maleated hyaluronic acid (MAHA) is widely used in biomedical applications due to its ability to undergo photocrosslinking, which allows for the formation of stable hydrogels under controlled conditions [103]. Synthesis of MAHA involves the esterification of the hydroxyl group on HyA with maleic anhydride (MA) (Fig. 1). This reaction can be carried out in various organic solvents such as DMSO, formamide, or water, which help open the ring of MA and facilitate the reaction with HyA [[104], [105], [106]]. Organic solvent-based reactions are currently the most effective, with degree of modification values reaching as high as 88.2 % when using formamide [103,104], but their environmental impact has driven the development of solvent-free alternatives. Organic solvent-free protocols, while more sustainable, yield varying DS, the average number of maleate groups attached per repeating unit of hyaluronic acid, values, and increased HyA degradation. For instance, microwave irradiation (50–200 W, 100 °C, 5 min) accelerates esterification but yields DS values of only 1.9–2.2 while significantly reducing HyA MW [105]. Similarly, conventional heating (110–120 °C, 5 min) achieves comparable DS (1.4–2.1) but suffers from high material losses and degradation. Adding bases like potassium carbonate (K_2_CO_3_) to solvent-free reactions enhances DS (up to 2.47) and yields, but HyA degradation remains a challenge. A notable low-temperature, base-assisted approach avoids heating entirely, producing high-MW MAHA with minimal degradation, though DS values are limited (0.71–1.15) [105]. These degradation effects reduce HyA MW and may compromise functional properties. Interestingly, the MW of HyA influences its reactivity: high MW HyA reacts more efficiently, achieving higher DS values, whereas low MW HyA is less reactive at room temperature [105].

Following the synthesis of MAHA, the crosslinking density can be further adjusted through photocrosslinking. For instance, a two-step crosslinking process that involves initial photocrosslinking with Irgacure 2959, followed by crosslinking with PEG-diacrylate, has been shown to improve hydrogel stiffness, stability, and resistance to degradation [103].

MAHA has been explored in cartilage tissue engineering, wound healing, and drug delivery [104,106,107].

### 3.6 Norbornene-modified hyaluronic acid

Norbornene is a cyclic hydrocarbon consisting of a cyclohexane bridged by a methylene [108]. This molecule has been widely investigated in biochemical research [[109], [110], [111]]. Due to its ability to form covalent bonds with thiol groups in the presence of a photoinitiator and light, norbornene-modified HyA (NorHA) can form a hydrogel using thiol reactions such as Michael addition and thiol-ene free radical addition. This distinct photoreactive property is particularly advantageous for patterning or dynamically tuning crosslinking density during or after gelation by varying NorHA concentration, crosslinker:NorHA molar ratio, and light exposure time, with degrees of modification of approximately 20 % [112]. Additionally, functional groups such as labelling dyes and cell adhesion peptides can be incorporated using the same reaction [109,111]. A norbornene group is typically grafted to the hydroxyl group of HyA in a standard esterification process using the TBA salt of HyA and the carboxylic acid of norbornene, achieving a degree of modification of 15.8 % of HyA repeat units functionalised with norbonenes (Fig. 1) [113]. Using DMAP as a catalyst and Boc2O as a direct reagent, TBA is removed from the HyA chain and norbornene is added. Although norbornene is not functionalised on the same site of the HyA unit as the TBA group, the presence of TBA protects the carboxylic acid group, creating a selective reaction. Recent advances have been reported in the synthesis of NorHA, eliminating the need for organic solvents, high temperatures, and anhydrous environments, significantly simplifying the generation of NorHA, while achieving degrees of substitution of around 20 % and as high as 48 % [114]. Using MES as a solvent and DMTMM as the catalyst, this process attaches 5-norbornene-2-methylamine to the carboxylic acid group in an amide coupling reaction.

Using NorHA, hydrogels with a wide range of biophysical and biochemical properties can be generated, including elastic moduli ranging from below 10 Pa to 100 kPa [112,114,115]. This degree of control over functionalisation and mechanical properties, combined with the biocompatibility of NorHA, allows for a wide range of possible applications in the biomaterials field. NorHA serves as a platform for synthesising complex cyclic structures, genetic encoding for protein labelling, and biocompatible spontaneous gelation agents [[109], [110], [111]].

### 3.7 Thiol-modified hyaluronic acid

Thiol-modified HyA (HA-SH) hydrogels can be crosslinked *in situ* through disulphide bond formation or using acrylate functionalised crosslinkers [116,117]. Notably, HA-SH is commercially available as Heprasil® (BioTime, Inc.), in a mixture with thiolated-heparin [118]. Thiol modification of HyA typically involves amidation of the carboxyl group to form amide bonds, with degrees of modification ranging from 9 % to 90 % (Fig. 1) [[119], [120], [121], [122]]. Similar to other modifications, the carboxylic group is activated using reagents like HOBt, NHS, *N*-hydroxysulphosuccinimide (sulpho-NHS), or 2-chloro-1-methylpyridinium iodide [123,124]. Subsequently, these activated groups are coupled with amino groups to form amide bonds using carbodiimide derivatives, such as EDC or carbonyldiimidazole [125]. In the case of HA-SH, cystamine dihydrochloride is often used [[119], [120], [121], [122]].

Thiol groups in HA-SH can be oxidized by DMSO to form disulphide bonds, creating HA-SH hydrogels that allow non-water soluble drug loading within the hydrogel mesh for sustained release *in vivo* [126]. Hydrogen peroxide (H_2_O_2_) is another oxidizing agent used to trigger disulphide bond formation and hydrogel formation using HA-SH [127]. Alternatively, polydopamine (PDA) can be used as a crosslinker in a HA-SH hydrogel. Thiol groups ae introduced to HyA *via* the EDC-NHS reaction between the carboxyl groups of HyA and the thiol groups from cysteine. Concurrently, the catechol groups in PDA ae oxidized to form quinone, which then reacts with HA-SH through Michael-type addition reactions [128].

In addition to disulphide bonds, the formation of metal-ligand coordination bonds also contributes to the crosslinking of HA-SH hydrogels. Reversible metal-ligand interactions enable self-healing and tuneable mechanical properties. Thiol groups in HA-SH bind metal ions such as Zn^2+^ or Fe^3+^, forming dynamic, self-repairing networks suitable for injectable and 3D-printable applications [129]. HA-SH is frequently applied in wound healing, cartilage regeneration, and 3D printing [[130], [131], [132], [133]].

### 3.8 Tyramine-modified hyaluronic acid

Tyramine-modified HyA (HA-Tyr) is widely used in biomedical applications due to its injectability, biodegradability and tuneable mechanical properties [[134], [135], [136]]. HA-Tyr-based hydrogels require a low DS, typically below 5 %, to form stable hydrogels [137]. HA-Tyr is synthesised by forming an amide bond between the carboxyl group of HyA and tyramine (Fig. 1). This modification is achieved using coupling agents such as EDC and NHS [138], with DMTMM offering enhanced control over substitution levels, e.g. 17.25 %, subsequent hydrogel properties, and tyramine stability at near-neutral pH [139]. Crosslinking of HA-Tyr hydrogels can be achieved enzymatically with horseradish peroxidase (HRP) and H_2_O_2_, providing precise control over crosslink density. While H_2_O_2_ influences the mechanical strength of the hydrogels, HRP regulates the gelation rate independently of each other [140]. Alternatively, HA-Tyr can be photocrosslinked using visible light and dyes such as riboflavin [141], rose bengal, or eosin Y [142].

Similar to other modified forms of HyA, HA-Tyr can undergo photocrosslinking. Loebel et al. found that enzymatic crosslinking (using HRP and H_2_O_2_) improved cell spreading and focal adhesion in MSCs compared to photocrosslinked hydrogels [143]. However, enzymatic crosslinking has two notable disadvantages: it can lead to undesirable mechanical degradation of HA-Tyr-based hydrogels over time, while residual H_2_O_2_ can reduce cell viability. To overcome these issues, a two-step gelation process was suggested, involving initial hydrogel preparation followed by the controlled addition of exogenous HRP. This method improved mechanical properties and cell viability while supporting the proliferation of encapsulated human adipose-derived stem cells (hADCs) [144].

HA-Tyr has been used in applications such as drug delivery, tissue regeneration, and 3D printing [[134], [135], [136]].

### 3.9 Sulphate-modified hyaluronic acid

Sulphation of HyA enhances its inherent bioactivity and improves hydrogel stability through the provision of a negative charge [145,146]. Sulphation enhances growth factor binding and the interaction of HyA with chemokines and cytokines to reduce inflammatory responses [[147], [148], [149]]. Furthermore, HyA is the only non-sulphated glycosaminoglycan (GAG), and the process of sulphation allows it to mimic other bioactive ECM components like heparan sulphate and chondroitin sulphate [147].

Addition of sulphate groups to HyA typically occurs using sulphating reagents, most commonly complexes of sulphur trioxide (SO_3_) with organic amines or amides, or divinylsulphone (DVS) [147]. SO_3_ is most commonly used in conjunction with dimethylformamide (DMF) for sulphation due to mild reaction conditions, resulting in minimal polymer degradation [150]. In this reaction, SO_3_/DMF forms an activated sulphate species, which then reacts with hydroxyl groups in the HyA backbone. This results in the formation of sulphate esters in the *N*-acetylglucosamine and glucuronic acid units of HyA (Fig. 1) [151]. The degree of sulphation can be easily adjusted with this method by altering concentrations of the SO_3_/DMF complex, achieving sulphur contents of 1.61 % and 2.85 % for low and high sulphated HyA respectively [150]. During this process, HyA is commonly used in its HA-TBA form to improve its solubility, to reduce polymer degradation, and to enhance the reactivity of the hydroxyl groups [151].

Another method commonly employed is modification using DVS. With this method, DVS reacts with hydroxyl groups on the polymer *via* Michael addition, allowing for a degree of modification ranging from 21 % to 89.25 % and achieving a sulphur content of 1.0–1.3 [152,153]. This method is advantageous as DVS acts simultaneously as a sulphate donor and crosslinker, permitting one-step modification of HyA (HA-DVS) and hydrogel formation [153]. However, this reaction is limited by the need for an alkaline reaction environment.

HA-DVS-based hydrogels have been developed for cartilage repair, drug delivery, and ophthalmic applications [150,[152], [153], [154]].

## 4 Functionalisation of hyaluronic acid with bioactive moieties

**Fig. 2:**
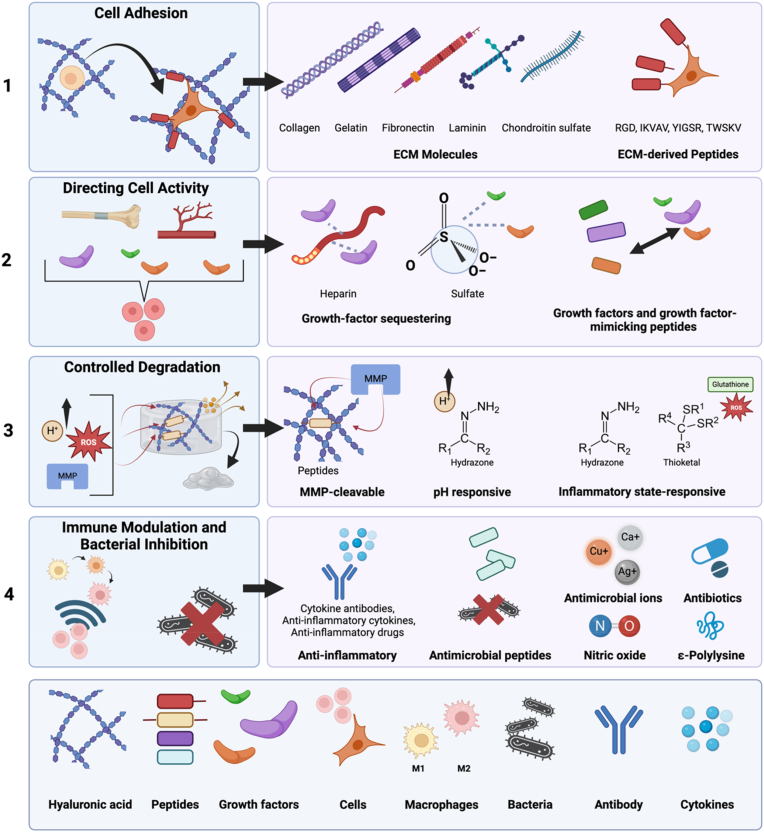
Graphical depiction of bioactive moieties that have been conjugated to enhance the bioactivity of modified HyA hydrogels to (1) support cell adhesion, (2) direct cellular activity, (3) control degradation and release kinetics, and (4) promote immunomodulation and antimicrobial activity. *Created in BioRender*. *Chang*, *Y*.*J*. *(2025)*https://BioRender.com/w40l932.

Modified HyA possesses many desirable properties as the polymeric backbone of hydrogel, with retention of its biocompatibility and biodegradability. These modifications also offer opportunities to conjugate bioactive moieties to drive specific cell responses, as well as additional functionalities including anti-inflammatory and antimicrobial activity. Common approaches to enhance the bioactivity of HyA hydrogels include the incorporation or conjugation of ECM molecules, bioactive peptides, and growth factors (Fig. 2).

Early approaches to HyA hydrogel functionalisation involved physically encapsulating bioactive molecules within the HyA network prior to crosslinking. For example, addition of bone morphogenetic protein 2 (BMP-2) or p15 peptide to HyA hydrogels enhanced osteogenic function of MSCs and osteoblast-like cells [62,155].

More recent approaches to modification allow for sustained release, or on-demand release in response to stimuli, such as pH or photostimulation [156,157]. These approaches involve specific chemical conjugation of bioactive moieties to HyA, allowing sustained activity and retention of bioactive molecules [158]. Conjugation of epidermal growth factor (EGF) to HyA for wound healing was accomplished through covalent binding of the amine groups in EGF to HA-CHO [158]. More than double the concentration of EGF was maintained *in vivo* at 4 h and 8 h compared to non-conjugated EGF, with EGF bioactivity maintained over 8 days in a preclinical wound model [158].

The sections below will describe common approaches developed to impart specific bioactivity to HyA-based hydrogels. We will describe approaches to enhance cell adhesion, direct cellular activity, control hydrogel degradation, and modulate immune responses.

### 4.1 Cell adhesion

HyA does not inherently possess cell adhesion or integrin-binding domains, and thus typically requires the addition of specific ligands to support cell adhesion [30,159]. This can be achieved by conjugating whole ECM molecules or ECM-derived peptides containing specific cell adhesion sequences. Common ECM molecules used include collagen, fibronectin, and laminin, while a wide range of peptide sequences have been identified that promote cell adhesion, growth, and proliferation, with the fibronectin-derived arginylglycylaspartic acid (RGD) sequence being the most widely used [113,160,161].

Conjugation of human-like collagen in a HA-Tyr hydrogel crosslinked with 1,4-Butanediol diglycidyl ether showed improved cell viability and adhesion of L929 line mouse fibroblasts, with a dose-dependent response [162]. Furthermore, inclusion of collagen also reduced the inflammatory response observed in preclinical mouse and rabbit models. A gelatin-HyA hydrogel modified with addition of methacryloyl groups to both gelatin and HyA was generated as a biomimetic ECM platform for cartilage growth [163]. The hydrogel was further functionalised with chondroitin sulphate and displayed enhanced chondrogenesis, improved cell morphology, and increased ECM deposition from encapsulated chondrocytes over 8 weeks.

Seidlits et al. demonstrated the importance of ECM-molecule incorporation within HyA hydrogels to maintain viability, proliferation, and promote tube formation by endothelial cells [157]. MeHA hydrogels were unable to maintain the viability of encapsulated human umbilical vein endothelial cells (HUVECs), whereas fibronectin-conjugated MeHA hydrogels sustained HUVEC viability and demonstrated significant cell spreading and network formation. Similarly, incorporation of laminin in a HA-SH hydrogel crosslinked with PEG-DVS significantly enhanced survival and proliferation of neural progenitor cells (NPCs) with little cell adhesion present in laminin-free hydrogels [164].

Incorporation of ECM-derived peptide sequences offers a number of advantages over whole ECM molecules, as synthesis of short sequences can be more cost-effective, and peptides allow more specific and defined conjugation reactions [165,166]. However, limitations of using peptide sequences must also be considered, including potentially reduced downstream signalling and altered integrin binding dynamics [167,168]. Nonetheless, conjugation of adhesion peptides is widely applied to support cell adhesion in HyA hydrogels. A cysteine-terminated RGD peptide was conjugated to 4-arm PEG-maleimide, which was subsequently used as a crosslinker to form a HA-SH hydrogel. RGD-decorated HyA hydrogels demonstrated enhanced NPC viability up to 70 days of culture in oligodendrocyte differentiation medium [169]. A co-culture of HUVEC and fibroblast in an HRP-crosslinked RGD peptide-decorated HA-Tyr hydrogel promoted *in vitro* cell adhesion and vascular tube formation, subsequently enhancing angiogenesis *in vivo* [136]. Adhesion peptides including RGD and YIGSR, derived from laminin motifs, was grafted to NorHA using terminal cysteines [113]. The selectivity of cell adhesion by peptides, combined with the distinct reactions between NorHA and each peptide, can create precise cellular organization through light-controlled patterning [113]. Another approach involves incorporation of multiple adhesion peptides in tandem to optimise cell adhesion and integrin activation. For instance, the impact of RGD, IKVAV, YIGSR, and TWSKV peptides on human salivary gland stem cells was evaluated in hydrogels based on HA-SH and AcHyA [170]. It was found that only the HyA hydrogels with RGD and IKVAV peptides improved cell attachment over non-peptide conjugated hydrogels. A similar study demonstrated that the presence of the IKVAV peptide, regardless of the presence of the RGD or YIGSR peptides, increased NPC spreading in NorHA hydrogels crosslinked with 4-arm PEG [171]. Evidently, the HyA hydrogel system provides the necessary versatility for incorporating various peptides to allow testing of numerous adhesion motifs, and their application in more complex systems. Furthermore, promotion of specific integrin-ECM ligand interactions or expression in cells has direct implications for cell cytoskeletal properties, such as influences over stem cell fate decisions or maintenance of cell naivety, and as such can be harnessed within model systems or developed tissues by specific patterning of adhesion motifs.

### 4.2 Directing cell activity

Small molecules, proteins, and growth factors are widely used to support or direct cell behaviour. However, their therapeutic relevance is limited by short half-lives and rapid diffusion into surrounding tissues [172]. Although growth factors themselves can also be conjugated to hydrogel materials, non-selective conjugation reactions can lead to sub-optimal bioactivity [173]. As such, a range of approaches have been developed using HyA-based hydrogels to selectively bind growth factors, either specifically or non-specifically, or by the conjugation of growth factor-mimicking peptides [174].

One of the most widely used molecules for sequestering growth factors is the highly-sulphated GAG heparin, due to it being a natural mechanism, lending to the abundance of heparin-binding domains present in growth factors [175]. Heparin has been covalently conjugated to hydrogels through carbodiimide chemistry, Michael-type addition, and thiol-maleimide chemistry [175]. Conjugation of high MW thiolated heparin *via* Michael-type addition in a peptide-crosslinked AcHyA hydrogel demonstrated enhanced retention of transforming growth factor (TGF)-β1, which supported robust differentiation of encapsulated cardiac progenitor cells (CPCs) into endothelial cells [176,177]. Furthermore, these heparin-containing AcHyA hydrogels could maintain the viability of encapsulated CPCs *in vivo* and supported anastomosis with the host vasculature [177]. Pike et al. varied the heparin concentration to assess its effect on vascular endothelial growth factor (VEGF) and basic fibroblast growth factor (bFGF) release from 3,3′-dithiolbis (propanoic hydrazide)-modified PEG-diacrylate-crosslinked HyA hydrogels [178]. VEGF and bFGF release were sustained for 42 days by conjugation of heparin at concentrations from 0.03% to 1%. Implanted hydrogels demonstrated enhanced vascularisation and increased microvessel density *in vivo* over 28 days. In contrast, heparin-free hydrogels failed to sustain microvessel proliferation beyond 14 days. In all, incorporation of heparin in hydrogels permits sequestration of a diverse array of heparin-binding growth factors, which are capable of maintaining bioactivity, while presenting these growth factors to cells in a natural way, leading to improved hydrogel functionality both *in vitro* and *in vivo*.

Modification of HyA with sulphate groups can similarly add growth factor-sequestering properties, through electrostatic interactions between the negatively-charged sulphate group and positively-charged amino acids in growth factors [[147], [148], [149]]. Purcell et al. reacted MeHA with a DMF-sulphate complex [179], which significantly reduced stromal cell-derived factor (SDF)-1α release rate over 12 days. The same effect was observed in a study where MeHA hydrogels were combined with sulphated HyA salts and photocrosslinked with Irgacure 2959 [150]. The sulphated hydrogels sequestered TGF-β1, showing a 40–50 % reduction in the rate of release compared to non-sulphated hydrogels over 7 days. However, it must be considered that this approach is most effective with heparin-binding growth factors, although non-heparin-binding growth factors have also demonstrated sequestration by sulphate groups through electrostatic interactions [156].

Alternatively, growth factor-mimicking peptides can be conjugated to HyA, similar to approaches used for cell adhesion peptides. Conjugation of the VEGF-mimetic peptide KLT to a 1,1′-carbonyldiimidazole-modified HyA hydrogel enhanced angiogenesis and reduced glial scar formation following implantation in a murine model of traumatic brain injury, compared to HyA-only hydrogels [180]. The prolonged availability of a BMP-2-mimetic peptide in a HyA hydrogel modified with Methyltetrazine-PEG_4_-amine hydrochloride and trans-cyclooctene-amine hydrochloride demonstrated enhanced support for the growth and differentiation of human dental pulp stem cells towards an osteogenic lineage [181]. Injection of a BMP-mimetic peptide CK2.1 conjugated to HyA nanoparticles in a mouse model of osteoarthritis demonstrated reduced osteoarthritic damage and enhanced cartilage repair, compared to HyA-only and sham treatments [182]. Furthermore, immunohistological staining indicated higher expression of collagen type II and IX in CK2.1-treated mice, indicating healthy cartilage regeneration. This study demonstrates the improved efficacy of BMP-mimetic peptide-conjugated HyA nanoparticles compared to standard approaches comprising unmodified HyA-only injections. Overall, peptide conjugation has demonstrated consistent efficacy in directing cellular activity throughout a range of cell types and delivery methods.

### 4.3 Controlled degradation and release

The degradation kinetics of HyA hydrogels can be controlled either to match the rate of cell growth to allow cell-mediated remodelling, or in response to stimuli. Controlled or tuneable degradation of HyA hydrogels can be accomplished using MMP-cleavable crosslinking peptides, which support cell-mediated degradation [183]. As the cell population expands, increasing amounts of MMPs are released, resulting in HyA hydrogel degradation co-ordinated with tissue formation. Culture of hMSCs in an AcHyA hydrogel using bis-cysteine-containing MMP-degradable peptide crosslinkers resulted in improved cell viability, robust spreading, elongation, and increased expression of chondrogenic markers [184]. This demonstrates the importance of the ability for the hydrogel to be modified by the cells to support their growth. Diseased tissue, notably cancers, are known to over-produce certain MMPs [185]. As such, MMP-degradable HyA-based drug carriers can lead to increased drug release specifically in diseased tissues. The efficacy of targeted and reduced drug dosages was observed in a study investigating HyA-coated triptolide drug nanoparticles in a mouse model of breast cancer [186]. HyA-coated delivery exhibited significantly lower systemic toxicity compared to free triptolide, owing to controlled degradation of the nanoparticles in diseased tissues with excess MMP.

Other disease states also lead to the overexpression of MMPs, which can be leveraged to control therapeutic release. For example, Purcell et al. developed a combination HA-CHO, aldehyde-dextran sulphate, and maleimide-modified HyA hydrogel conjugated with an MMP-inhibitor to target MMP-over-expressing regions in a model of myocardial infarction [187]. Implantation of the HyA hydrogels in pig models demonstrated localised MMP inhibition and improved heart function, such as left ventricular ejection fraction and chamber dilation, following induction of myocardial infarction compared to negative and drug-free controls.

In addition to increased MMP expression, another feature of diseased tissues, including tumours, is acidosis, leading to the development of pH-responsive drug carriers [188,189]. This was demonstrated using doxorubicin (DOX)-conjugated HyA nanoparticles crosslinked with hydrazone bonds, which are cleaved under acidic conditions thus releasing their drug cargo [190]. The HyA nanoparticles demonstrated more than a 5-fold release of DOX at pH 5 compared with pH 7.4, and successful targeting of cancerous cells, with a 60 % decrease in viability compared to healthy cells.

HyA-based hydrogels and nanoparticles have also been developed to degrade in response to inflammatory mediators, which are produced in response to oxidative stress and inflammation [191]. Examples include a dual hydrazone and photocrosslinked HyA hydrogel developed as a glutathione-responsive carrier, resulting in the expansion of encapsulated MCF-7 cancer cell line, providing a platform to model *in vivo* tumorigenicity [191]. A ROS-responsive hydrogel carrier of probiotics was generated for the treatment of colitis, through synthesis of a MeHA hydrogel crosslinked with a thioketal crosslinker [192]. The hydrogel carriers were selectively degraded by excess ROS present at the disease site, targeting the delivery of probiotics to sites of inflammation. The HyA hydrogel prolonged probiotic activity *in vitro* and reduced inflammation when implanted in a preclinical model of colitis.

### 4.4 Immune modulation and bacterial inhibition

HyA is known to play different roles in inflammation depending on its MW, with high MW HyA identified for its anti-inflammatory properties [193]. A 4-arm PEG amine-crosslinked high MW HyA hydrogel demonstrated protective, anti-inflammatory effects on nucleus pulposus cells by preventing extended inflammation through CD44 receptor binding and suppression of inflammatory receptors interleukin (IL)-1R1 and MyD88 [194]. This known anti-inflammatory effect can be further enhanced with functionalisation and the addition of anti-inflammatory molecules including antibodies, cytokines, and anti-inflammatory drugs, while antimicrobial function may also be introduced by the addition of molecules like antimicrobial peptides (AMPs), ions, and antibiotic drugs.

Conjugation of an anti-tumour necrosis factor (TNF)-α antibody to HyA using EDC, sulpho-NHS, and DMAP reduced the damaging effects of chronic inflammation in a pre-clinical wound model and provided sustained modulation of the inflammatory response [195]. Delivery of anti-inflammatory cytokines have also demonstrated success for immunomodulation [196]. Conjugation of the anti-inflammatory cytokine IL-10 to a HA-SH and thiolated gelatin (Gel-SH) hydrogel modified with heparin and crosslinked with PEG-diacrylate suppressed fibrosis in lung fibroblasts *in vitro* and in pre-clinical models of pulmonary fibrosis. A similar effect has also been observed with anti-inflammatory drugs. Carboxymethylated HyA conjugated to amine-modified ibuprofen demonstrated a reduction in expression of proinflammatory cytokines and reduced cytotoxicity compared to free ibuprofen in mouse macrophages [197].

AMPs support wound healing by preventing bacterial infection of chronic wounds [198]. Chemical conjugation of ε-Polylysine (EPL) to HyA nanofibers enhanced antimicrobial activity by allowing its sustained release and surface area. Agar diffusion assays demonstrated that areas covered by EPL-conjugated HyA mats were completely free of *Escherichia coli* and *Staphylococcus aureus* bacteria after 24 h and a zone of inhibition around the mat [199]. Immobilisation of the AMP Tet213 conferred antimicrobial properties to an alginate-HyA-collagen wound dressing [200]. Addition of Tet213 not only increased the rate of wound closure, but also halved the amount of bacteria present compared with gauze-only and Tet213-free dressings.

Further approaches to confer antimicrobial properties to hydrogels include conjugation or physical crosslinking with ions possessing antimicrobial properties, such as copper, calcium, or silver [201]. For example, Cu(II) cations used to crosslink hydrazide-HyA hydrogels demonstrated *in vitro* cytocompatibility with mouse fibroblasts, whilst eradicating *Escherichia coli* and *Staphylococcus aureus* colonies on agar plates [202]. Antimicrobial properties have also been conferred to HyA hydrogels by the addition of nitric oxide-releasing nanofibers, as nitric oxide has inherent biocidal activity [203]. Nitric oxide-releasing nanofibers were incorporated by the conjugation of nitric oxide donor N-diazeniumdiolate modified with N-methylethylenediamine to MeHA-TBA through EDC-NHS chemistry [204]. Conjugation to HyA allows the synthetic fibres to degrade *in vivo*, which reduces toxicity. Conjugation of antibiotics has demonstrated efficacy in overcoming resistance developed in some antibiotic-resistant bacteria strains, thereby maintaining clearance of bacteria in wound sites. Zhang et al. demonstrated the conjugation of ciprofloxacin using 1,1′-carbonyldiimidazole to HyA particles crosslinked with glycerol diglycidyl ether are capable of tuneable degrees of antibiotic delivery over a period of 1 week [205].

## 5 Applications of hyaluronic acid-based hydrogels

Owing to the wide range of modifications and functionalisation strategies, HyA hydrogels have been used in a wide variety of applications in regenerative medicine. This includes the development of 3D *in vitro* models of healthy and diseased physiology, as well as for the repair and regeneration of soft and hard tissues alike (Table 1).

**Table 1:** Selected *in vitro* studies using HyA-based hydrogels.

Application	Hydrogel composition	Crosslinking strategy	Functionality	Cell model	*In vitro* effects	Reference
Stem cell culture	MeHA	Irgacure 2959	Ability of HyA to maintain stemness of hESCs	hESC	Encapsulated hESCs maintained pluripotency Cells released from HyA maintained viability and pluripotency Encapsulated cells retain ability to differentiate in 3D	[208]
Cartilage tissue engineering	HA-Tyr	H_2_O_2_, HRP	Effect of substrate stiffness on differentiation and chondrogenesis	Goat MSC	Increasing substrate stiffness and crosslinking was associated with fibrous phenotypes of MSC differentiation	[212]
Cartilage tissue engineering	MeHA GelMA	LAP, stereolithographic bioprinting	Influence of cell density and hydrogel composition on cartilage development	Porcine chondrocytes encapsulated in the bioinks	Different cell densities and hydrogel compositions modulate ECM production and chondrocyte behaviour	[232]
Central nervous system	NorHA	LAP, DTT	OPC proliferation, metabolic activity, and morphology in response to stiffness	OPCs	Larger cell spheroids formed in lower stiffness hydrogels with larger mesh sizes Lower stiffness hydrogels supported improved OPC metabolic activity and growth compared to stiffer hydrogels	[233]
Skin tissue engineering	HyA Agarose Collagen I Dermatan sulphate Elastin	Thermosensitive and pH-sensitive gelation	Development of advanced bilayered skin substitute engineering	HDFs (top layer) hMSCs (bottom layer)	Bilayered structure and cell viability maintained for 21 days ECM production by human dermal fibroblast hMSC adipogenesis	[234]
Cancer research	AcHyA-RGD	MMP-cleavable crosslinker	Effect of hydrogel stiffness and oxygen on cancer cell spreading	Human fibrosarcoma cell line	Normoxic conditions and stiffened substrates inhibited cell sprouting and spreading Cells spread regardless of substrate stiffness in hypoxia	[66]
Cancer research	MeHA-RGD	DTT	Effect of stiffness and RGD density for glioblastoma invasion	Human or rat glioblastoma	Increased stiffness and peptide density increased glioma adhesion and invasion	[215]
Cancer research	NorHA-RGD	VPM or DTT, Irgacure 2959	Effect of matrix on tumoroid growth	Human oesophageal adenocarcinoma cells	Increased hydrogel stiffness enhanced tumour cell proliferation and expression of oesophageal adenocarcinoma cell-associated genes	[218]
Cancer research	Gelatin-HyA	LAP	Effect of EGFR mutation status on erlotinib efficacy	Human glioblastoma cells	Cells possessing EGFRvIII mutation were resistant to erlotinib treatment	[221]
Cancer research	MeHA	Irgacure 2959	Development of ECM-mimicking 3D breast tumour models to replicate the native tumour environment	MCF-7 breast cancer cells	Facilitated MCF-7 cell proliferation and aggregate growth Enhanced malignancy of MCF-7 cells compared to 2D culture Promoted greater invasion and tumorigenesis capabilities of MCF-7 cells in 3D Mimicked *in vivo* tumour phenotypes more closely than 2D culture	[235]
Central nervous system	MeHA	Irgacure 2959	Comparing healthy and Down Syndrome derived neurons hiPSC differentiation and maintenance in 3D	hiPSC	Single-cell encapsulation detrimental for neurite outgrowth Soft hydrogel promotes neuronal phenotype and extended neurite growth Soft hydrogels unable to promote neuronal phenotype in Down Syndrome-derived cells	[223]
Neuroinflammation modelling	Collagen I-HyA-PEG diacrylate	Collagen I, PEGDA	Impact of HyA concentration on astrocyte growth	Human astrocytes	Decreased HyA concentration increases inflammatory response Astrocyte length decreases with reduced HyA content	[224]
Central nervous system	MeHA	N-vinylpyrrolidone Eosin Y Triethanolamine	HyA as model for electrode biocompatibility	Rat astrocytes, rat microglia	Microglia engulfed other cell types Microglia and astrocyte response to electrode insertion recapitulated glial scarring observed *in vivo*	[226]

GelMA: gelatin-methacrylate; HDFs: human dermal fibroblasts; hiPSC: human induced pluripotent stem cell-derived; LAP: lithium phenyl-2,4,6-trimethylbenzoylphosphinate; OPCs: oligodendrocyte progenitor cells; PEGDA: poly(ethylene glycol)-diacrylate.

### 5.1 HyA hydrogels as *in vitro* model systems

The development of accurate *in vitro* models is essential to further the understanding of disease pathologies and mechanism of tissue repair [206,207]. However, one crucial consideration for the development of these models is the provision of a suitable 3D matrix to more faithfully recapitulate the *in vivo* microenvironment. HyA has emerged as a suitable candidate owing to its range of functionalisations and its tuneability to mimic native tissue ECM.

HyA-based hydrogels have been demonstrated to support maintenance of embryonic stem cells, notably seen in the work of Gerecht et al., where MeHA hydrogels were used to maintain the undifferentiated state of human embryonic stem cells (hESCs) [208]. This was attributed to the known ability of HyA *in vivo* to coregulate factors including the proliferation, adhesion, and morphogenesis of hESCs (Table 1) [209]. The tuneable mechanical properties of HyA-based hydrogels can also control the differentiation of adult stem cells and progenitors [54,209,210]. For example, stiff HyA substrates enhanced the spreading of MSCs and temporally affect differentiation; early substrate stiffening after 1 day of culture resulted in cells committing towards an osteogenic lineage [54,209,210]. The tuneability of HyA makes it uniquely suitable to support dynamic studies like these, making *in vitro* systems more capable of recapitulating ECM mechanics in disease, such as in osteoarthritis. Studies also compared the effect of the stiffness of HyA-based substrates on MSC maintenance and differentiation towards chondrogenic lineages [211,212]. HA-Tyr hydrogels crosslinked to varying degrees with H_2_O_2_ and HRP were used to investigate the effect of stiffness on chondrogenic differentiation of MSCs. It was found that MSCs exhibited enhanced chondrogenesis in substrates of lower stiffnesses, whereas MSCs shifted towards a more fibrous phenotype on stiffer substrates with increased crosslinking (Table 1).

Modular HyA hydrogels can also be used to study disease progression, by isolating individual attributes like degradation and substrate stiffness. HyA is uniquely suitable for cancer studies and treatment applications due to its high prevalence in the tumour microenvironment and its interaction with the CD44 receptor in many cancer types [213]. Dickinson et al. used microcontact printing and silane chemistry to micropattern RGD-modified and MMP-cleavable peptide crosslinked AcHyA and fibronectin on a glass substrate to direct the growth of breast cancer cells and endothelial colony-forming cells (ECFCs) in a patterned co-culture [214]. Fibronectin-treated HyA-patterned substrates permitted controlled regions of cell adhesion and inhibition, and mimicked cancerous ECM by the abundance of interactions with CD44 receptors. ECM components deposited by breast cancer cells influenced the growth of ECFCs, and subsequently capillary formation and vasculogenesis. Shen et al. sought to determine the effect of substrate stiffness and oxygen levels on fibrosarcoma cell growth using an AcHyA hydrogel [66]. It was demonstrated that oxygen tension had a greater impact on cell fate and endothelial invasion than substrate stiffness. Under normoxic conditions, increased hydrogel stiffness inhibited both cell sprouting and spreading, however no stiffness-mediated inhibition was observed in hypoxic conditions (Table 1). Similarly, Ananthanarayanan et al. compared the influence of hydrogel stiffness and RGD adhesion peptide density on rat and human glioblastoma invasion using a MeHA hydrogel crosslinked with DTT [215]. Increasing HyA hydrogel stiffness and peptide density enhanced glioma cell adhesion and consequently tumour cell invasion (Table 1). Conjugation of an RGD peptide to a MeHA hydrogel crosslinked with a protease-degradable or non-degradable peptide sequence was used to elucidate the role of TGF-β in glioma cell invasion, by uncoupling the effect of RGD and matrix degradability [216]. TGF-β-stimulated invasion was found to increase in HyA hydrogels containing RGD, but not in identical hydrogels lacking RGD, indicating its necessity for the TGF-β-mediated pathway of invasion.

HyA hydrogels have been used to develop patient-specific 3D cancer models by encapsulation of cells collected from patients, termed “tumoroids” [217]. Tumoroids provide a more accurate *in vitro* model of disease, serving as a bridge between *in vitro* drug testing, clinical trials, and personalised medicine. Cruz-Acuña et al. encapsulated patient-derived oesophageal adenocarcinoma tumoroids in NorHA hydrogels of varying stiffnesses to investigate the impact of ECM mechanics on patient-derived EAC tumoroids [218]. These HyA hydrogels had covalently-incorporated RGD peptides and were crosslinked with protease-degradable GCNSVPMSMRGGSNCG (VPM) or non-degradable DTT. It was found that increased stiffness enhanced EAC progression, with a higher percentage of proliferating cells and expression of EAC-associated genes in tumoroids, including CD44 (Table 1). Furthermore, tumoroid models have demonstrated functionality for tailored drug screening on resistant cancer types by expansion of patient-derived diseased cells in HyA-based systems [219,220]. A HyA-based hydrogel was developed to evaluate the efficacy of the cancer drug erlotinib for the treatment of mutated derivations of glioblastoma, using patient-derived tumour cells [221]. The HyA-based hydrogel was tuned to match native neural tissue, and it was found that EGF receptor (EGFR) mutation status impacted drug efficacy, with no cytotoxicity observed in EGFRvIII glioblastoma cells (Table 1). A similar approach using an AcHyA and HA-SH based tumoroid encapsulating a patient-derived prostate cancer cell line was used to probe the efficacy of DOX-loaded nanoparticles for cancer treatment [217]. The HyA hydrogel's mesh size was matched to known values for cancerous tissue, and the drug was found to diffuse effectively throughout the cancerous matrix. Overall, the platform determined tumoroids have enhanced resistance to DOX compared to the same cells in 2D, highlighting the importance of 3D-based platforms for disease modelling. A hepatocyte carcinoma cell line maintained in 3D culture on a HyA-poly(methylvinylether-*alt-*maleic acid) hydrogel was used to investigate the antitumour mechanism of cisplatin [222]. The use of a HyA-based hydrogel was crucial in mimicking the *in vivo* tumour environment, as demonstrated by enhanced cell metabolism and the liver-like morphology of the encapsulated cells (Table 1). Evaluation of cell behaviour revealed that cisplatin exerts its effect through DNA fragmentation, reorganisation of actin filaments, and through action on the mitogen-activated protein kinase pathway. In all, HyA-based hydrogels have demonstrated success in elucidating the physiology of cancer cells through its tuneability and capacity to mimic the ECM microenvironment.

HyA-based hydrogels have also been used to study mechanisms of a range of other diseases, such as those involving the central nervous system (CNS). The effect of stiffness on cell growth and differentiation has been studied with NPCs. In a MeHA hydrogel, NPCs from individuals with Down Syndrome had a varied expression of markers of maturation and differentiation compared to healthy NPCs, and their growth on softer hydrogels promoted neural differentiation [223]. A HyA-collagen-PEG hydrogel with encapsulated astrocytes was used to better understand the impact of reduced HyA content *in vivo*, as this has been implicated in many neurodegenerative diseases [224]. Hydrogels with lower HyA content were posited to model disease progression by the increased expression of inflammatory markers like nitric oxide synthase, IL-1β, and TNF-α. Hydrogels low in HyA were found to reduce astrocyte processes and increase reactive and inflammatory markers, mimicking disease occurrences (Table 1) [224]. HyA-based hydrogels were also used to generate a microfluidic *in vitro* model of Alzheimer's disease to elucidate the interactions and effects of monomeric and oligomeric β-amyloid plaques in disease pathophysiology [225]. Human astrocytes were cultured in a 3D microfluidic platform incorporating a HA-SH hydrogel with collagen I and fibronectin. The microfluidic system could recapitulate interstitial fluid and served to deliver either monomeric or oligomeric β-amyloid to recapitulate *in vivo* disease progression. Due to the physical and compositional similarity to CNS tissue, MeHA hydrogels have been developed to interrogate factors influencing scarring from electrode insertions in the CNS. Microglia cultured on a photocrosslinked MeHA hydrogel platform appeared to engulf other encapsulated astrocytes, and both microglia and astrocytes reacted towards microwire insertion in a similar manner to what has previously been observed *in vivo* (Table 1) [226].

The tuneability of HyA-based hydrogels has also been harnessed to develop cell-based therapies. A HyA-coating was developed to extract and examine the impact of ADSC-derived extracellular vesicles (EVs) to restore homeostasis in osteoarthritic patient-derived synoviocytes [227]. HyA was key in this application not only for the isolation of EVs with enhanced CD44 levels, but for its cell interactions enhancing EV uptake. Furthermore, HyA-based hydrogels have been used to assess potential therapies such as autologous transplantation [228,229]. Commercial products such as HyAFF/Hyalograft allow expansion and transplantation of patient cells to repair cartilage and wound defects, demonstrating enhanced regeneration in long-term studies of autologous transplants [230,231].

Overall, HyA is uniquely useful as a biopolymer for furthering our understanding of both healthy and diseased cell mechanisms, owing to its biocompatibility and bioactivity, together with its range of hydrogel properties and tuneability to mimic the *in vivo* microenvironment. Not only has it allowed an increased understanding of the impact of uncoupled ECM properties on stem cell maintenance, but also the effect of biophysical and biochemical properties of ECM on cellular interactions, and drug efficacies in diverse applications including cancer, musculoskeletal, and neurodegenerative diseases.

### 5.2 HyA hydrogels as therapeutic interventions

HyA-based hydrogels offer significant potential in regenerative medicine due to their versatility and ability to be tailored for specific tissue applications. Their inherent biocompatibility, biodegradability, and capacity to mimic the native ECM make them suitable for a wide range of therapeutic interventions, as has been described in sections 3, 4. HyA hydrogels can be precisely modified to control properties such as stiffness, degradation, and bioactivity, facilitating the support of crucial cellular processes like adhesion, migration, and differentiation. HyA hydrogels aid in tissue regeneration by enhancing cell viability, promoting tissue integration, and supporting structural repair, while also addressing challenges like irregular defect sizes and shapes, and limited donor tissue availability [[236], [237], [238]]. Moreover, HyA hydrogels have proven effective in improving cellular interactions and inflammation control [26,239,240]. HyA-based hydrogels have been applied to various tissue types including bone, cartilage, muscle, skin, and neural, which will be discussed in the following sections.

#### 5.2.1 Cartilage

MeHA is widely used in cartilage applications. Usually photocrosslinked, this injectable HyA-based hydrogel can retain its properties and the viability of transplanted viability after extrusion [241], promoting cartilage-like matrix deposition after subcutaneous implantation in nude mice, or enhanced healing versus untreated control in a rabbit condyle defect model. MeHA can also be combined with other adhesive HyA-based materials, such as *o-*nitrobenzyl-grafted HyA to enable *in situ* crosslinking and achieve better fixation [242]. A chondrocyte-laden version of this HyA hydrogel showed remarkable integration of regenerated tissue with the native cartilage compared to the cell-free version in a swine knee arthroscopy model (Table 2).

**Table 2:** Selected preclinical studies using HyA-based hydrogels as therapeutic interventions.

Target tissue	Hydrogel composition and crosslinking strategy	Functionalisation	In vitro analysis	Preclinical model	Preclinical outcomes	Reference
Cartilage	HyA with di-amine crosslinkers	n/a	n/a	Rabbit condyle model	Fibro-cartilage deposition observed versus no healing on empty control	[273]
Cartilage	MeHA Photocrosslinking	n/a	n/a	Mice subcutaneous implantation	Increased chondrocyte count and ECM deposition 6 and 12 weeks after implantation	[274]
Cartilage	MeHA HANB-GL Photocrosslinking	n/a	n/a	Mice subcutaneous implantation Swine arthroscopy	HANB-GL groups showed higher ECM deposition in the subcutaneous model, while cell laden MeHA and HANB-GL double network gels showed better cartilage repair six months after swine arthroscopy compared to cell-less groups	[242]
Cartilage	HA-Tyr with gelatin Enzymatic crosslinking (tyrosinase)	EGCG anti-inflammatory compound	Attenuated catabolic gene expression in osteoarthritis chondrocytes in hydrogels with higher EGCG concentration after 2 days Increased matrix deposition in higher concentration hydrogels	Rat osteoarthritis model	Higher cartilage regeneration Lower expression of cartilage catabolic genes (collagen 1 and collagen X)	[243]
Cartilage	HA-VS Gel-SH Thiol-Michael addition	n/a	Significantly increased anabolic gene expression in BMSCs when compared to 2D 9 days after encapsulation	Mice subcutaneous injection	Increased tissue formation and lower CD31 and Collagen X expression	[244]
Cartilage	HA-Tyr Collagen I-tyramine Enzymatic crosslinking (HRP)	TGF-β1	Upregulation of anabolic genes in BMSCs both 14 and 28 days after encapsulation when compared to day 0	Rats with full thickness articular cartilage defect	Improved cartilage regeneration 8 weeks after implantation	[245]
Osteochondral	MeHA-GelMA photocrosslinked top layer β-TCP bioceramic bottom layer	n/a	n/a	Rabbit osteochondral defect	Enhanced GAG and tissue reconstruction compared to blank defects 12 weeks after implantation	[246]
Osteochondral	Photocrosslinked MeHA top layer GelMA-CaCl_2_ bottom layer	Nutrient-deprived BMSCs EVs and a silencer of VEGF/thrombin	Increased collagen 2 deposition and expression compared to non-starved and non-functionalised groups in bottom layer and increased alkaline phosphatase deposition in top layer	Rabbit osteochondral defect	Significantly higher defect filling and native-like tissue deposition in both subchondral and cartilage layer compared to empty control after 8 weeks	[247]
Osteochondral	HA-SH Thermal gelation and reaction between thiol and vinyl sulphone groups	Biodegradable and thermosensitive vinyl sulphonated triblock copolymer	Residence time of 32 days	Mouse model of osteoarthritis	Increase in bone mineral density Partial restoration of cartilage Reduced inflammation Decreased osteoclast maturation Reduction of pro-inflammatory mediators and cytokines	[275,276]
Bone	Photocrosslinked double network of MeHA and arginine-based poly (ester amide)	n/a	Increased mineralized area compared to 2D control	Rat calvarial defect	Increased osteogenesis in the defect compared to empty controls	[250]
Bone	Photocrosslinked MeHA encapsulating HyAp-PCL 3D printed framework	MSC-derived exosomes	Increased proliferation, migration and tube formation of endothelial progenitor cells in 24h compared to blank cultures	Rat calvarial defect	Increased bone deposition and osteogenic gene expression in the defect after 8 weeks compared to empty control	[251]
Bone	HyA-pyrogallol crosslinked through oxidation using calcium phosphates	BMP-2	Increased mineralization and osteogenic gene expression in hADSCs after 21 days	Mice calvarial defect	Significantly increased bone regeneration and collagen deposition compared to untreated defects	[252]
Bone	Tyr-HyA covalently crosslinked through oxidation (HRP)	GHK-Cu^2+^ and laponite	Hydrogel supported hMSCs with low cytotoxicity after 7 days	Rabbit maxillary sinus floor	Implant showed comparable performance of tissue regeneration to the commercially available positive control	[253]
Bone	MeHA Photocrosslinking	rhAm	Non-toxic and biocompatible Enhanced hPDLC differentiation Up-regulation of osteogenesis-related genes	Rat calvarial defect	Promoted bone regeneration Nearly complete defect repair, dense and uniform bone structures	[277]
Muscle	HA-SH crosslinked with PEGDA	Laminin-111 Minced muscle grafts	n/a	Rat TA VML injury model	Improved neuromuscular strength Reduced macrophage reaction Restored satellite cell content	[254]
Muscle	AcHyA Acrylate-thiol reaction with MMP-13 cleavable peptide (CQPQGLAKC)	bsp-RGD(15) peptide heparin	n/a	Rat TA VML injury model	Robust functional recovery Native-like vascularisation	[255]
Muscle	AcHyA Acrylate-thiol reaction with MMP-13 cleavable peptide (CQPQGLAKC)	bsp-RGD(15) peptide heparin	n/a	Rat using a novel model of masseter VML	Reduced fibrosis and defect size Greater FCSA	[256]
Muscle	HA-SH Collagen I	Collagen I PCL-MLT electrospun membranes	Supported C2C12 cells proliferation, migration, and viability; C2C12 cells proliferated and elongated better on PCL-MLT scaffolds Reduced intracellular ROS levels, protecting cells from stress Myogenic differentiation markers higher on PCL-MLT scaffolds PCL-MLT better supported cell growth and differentiation	VML rat model	Promoted vascularisation, adhesion sites for C2C12 cells Induced proliferation and differentiation of C2C12 cells	[257]
Muscle	HA-CHO AHA Hyaluronic acid-graft-PANI Acyl hydrazone bond formation between the aldehyde group of HA-CHO and the amino group of AHA	Leucine	Cytocompatibility 3D culture support for C2C12 and hADSC cells Enhanced cell recruitment and myogenic differentiation with Leu addition Enabled sustained cell delivery	Rat skeletal muscle injury	Degradable Enabled sustained cell delivery Promoted skeletal myogenesis and myogenic differentiation-related gene expression	[258]
Muscle	Ester of HyA Photocrosslinking	Satellite encapsulated cells	n/a	Wild-type mice Green fluorescent protein transgenic mice	Successful delivery of cells to the injury site Formation of new myofibers Development of neural and vascular networks Regeneration of the injured tissue	[259]
Muscle	HA-SH Thiolated chondroitin sulphate Acrylate-thiol with PEGDA	n/a	High cell viability and enhanced cell growth of encapsulated C2C12 cells Promoted myoblast proliferation and differentiation and increased expression of myogenic differentiation markers (MyoD, MyoG, MYH8)	Murine quadriceps VML model	Supported integration of implants De novo regeneration of skeletal muscle tissue *via* Pax7^+^ satellite cell migration Supported neovascularisation, myofiber formation Improved innervation Reduced scar tissue formation	[278]
Skin	MeHA MAHA Photocrosslinking	n/a	MeHA enhanced cell viability and proliferation of HUVECs, HDFs, and HAD-MSCs compared to MAHA Macrophage proliferation (MeHA) Higher IL-6 secretion (MeHA) Anti-inflammatory properties with high IL-10 levels	BALB/c mice	MeHA showed superior cell proliferation support while MAHA demonstrated a strong angiogenic response Both hydrogels achieved completed healing within 14 days	[107]
Skin	MeHA Dextran β-CD Photocrosslinking	Resveratrol pDNA-VEGF	No cytotoxicity High cell survival within the hydrogels VEGF expression in HUVECs significantly higher with Gel-Res/pDNA-VEGF scaffolds compared to other groups at days 3 and 7 pDNA-VEGF effectively retained its integrity	Burning induced splinted excisional wound model	Inhibited inflammation Promoted microvascular formation Controlled release of resveratrol and VEGF Increased VEGF secretion	[260]
Skin	MeHA GelMA Photocrosslinking	ADSCs	High cell viability and mobility ADSCs proliferation in hydrogels similar to 2D culture, with increasing DNA content over 21 days Spreading, elongation, and formation of networks from ADSCs in hydrogels Greater endothelial sprouting and blood vessel formation with hydrogels loaded with ADSCs or VEGF	Pathogen-free fertilized white leghorn chicken eggs (Gallus gallus domesticus)	Increased vascularisation	[261]
Skin	HA-CHO Hydrazide-modified sodium hyaluronate Aldehyde-modified cellulose nanocrystals Acylhydrazone crosslinking	PRP	Sustained release of PDGF, VEGF, and EGF, with a slower release after 24 h Over 90 % cell viability, with significant increase cell proliferation by day 3 High cytocompatibility Replacement every 3 days needed to prevent potential toxicity	Rat full-thickness skin defect model	Sustained PRP released Promoted granulation tissue formation Facilitated collagen deposition Accelerated re-epithelialisation and neovascularisation	[83]
Skin	HA-CHO HA-ADH Collagen “Double H-bonds” (hydrogen bond and hydrazone bond)	Metformin	Enhanced fibroblast adhesion and infiltration Inhibited macrophage growth Promoted fibroblast migration and collagen production	Diabetic mice wound model	ECM remodelling	[263]
Skin	HA-CHO Thiol-modified γ-PGA Thiol-aldehyde reaction	Antioxidant cysteine	High biocompatibility, with most 3T3 cells viable after five days and minimal cytotoxicity Cytocompatibility Safe for wound dressings	Rat full-thickness skin defect model	Promoted angiogenesis and collagen deposition	[79]
Skin	HA-Tyr Chitosan **Enzymatic crosslinking**	ACuBG nanoparticles	High viability and proliferation of L929 cells, with over 97 % viability after 24–48 h and increased cell density by day 7 Proliferation of L929 and HUVECs Higher strength and stiffness gels (GEL-3, GEL-4) promoted greater L929 cell migration Proangiogenic effects of Si and Cu ions induced the most HUVEC tube formation	Mouse model of full-thickness skin defects	Fully restored skin defects Growth of hair follicles and sebaceous glands within two weeks	[264]
Neural tissue	HA-SH Crosslinking with PEGDA	Gelatin Maleimide-functionalised PCL nanofibres	Survival, proliferation, and neuronal differentiation of NSCs	Rat model of spinal cord contusion	Prevented spinal cord thinning Promoted cell/tissue infiltration High cell density at injury site Supported a higher M2/M1 macrophage ratio (pro-regenerative macrophage polarization) Enhanced axonal growth	[267,268]
Neural tissue	MeHA Photocrosslinking	Ventral midbrain-derived NPC encapsulated cells	Differences in NPC phenotype based on hydrogel stiffness Spreading and elongation of spinal astrocytes Levels of GFAP dependent of hydrogel stiffness	Rat model of spinal dorsal hemisection injury	Reduction of astrocyte proliferation and CSPG deposition with high MW Reduced inflammation after acute spinal cord injury Reduced GFAP expression	[269,270]
Neural tissue	AcHyA Acrylate-thiol reaction with MMP-cleavable peptide (GCRDGPQGIWGQDRCG)	K-peptide (FKGGERCG) Q-peptide (NQEQVSPLGGERCG), and RGD (RGDSPGERCG)	n/a	Mouse stroke model	Reduced inflammation Astrocyte and NPC infiltration Enhanced vascular density NPC migration guided by astrocytes	[265]
Neural tissue	AcHyA Acrylate-thiol reaction with MMP-cleavable peptide (GCRDGPQGIWGQDRCG)	RGD, IKVAV, YIGSR peptides BDNF, BMP-4 Heparin iPS-NPC encapsulated cells	Promoted iPS-NPC survival and differentiation Axonal sprouting	Mouse stroke model	Improved cell survival Astrocytic differentiation in stroke cavity Increased GFAP and S100-β expression Promoted angiogenesis	[266]
Neural tissue	HyA-cysteamine dihydrochloride Thiol-disulphide exchange	Polypyrrole	Good cytocompatibility Promoted Schwann cell viability and cell marker expression (S100-β) Enhanced expression of nerve regeneration-related genes (MBP, NGF, S100-β)	Rat stroke model	Recovery of sciatic nerve function Reduced muscle atrophy High gastrocnemius muscle recovery rate Optimal nerve remyelination and function High β-III tubulin expression and glial cell marker S100-β High myelination and axonal regeneration	[272]
Neural tissue	HyA ADH-EDC crosslinking	PLGA microspheres containing BDNF and VEGF	BDNF protected neurons from glutamate-induced toxicity and improved cell survival VEGF promoted endothelial cell proliferation Proliferation of NSCs and formation of networks and extending neurites	n/a	See [279]	[280]
Neural tissue	HyA modified with PLL and anti-Nogo receptor antibody ADH-EDC crosslinking	PLGA microspheres containing BDNF and VEGF Anti-Nogo receptor antibody	n/a	Rat model of spinal cord injury	Improved locomotor recovery Integration of scaffolds with tissue Reduced inflammation Promoted angiogenesis and neural regeneration	[279]

HA-VS: sulphonated; hPDLC: human periodontal ligament cell; GFAP: glial fibrillary acidic protein; HANB-GL: hyaluronic acid with o-nitrobenzyl and gelatin; iPS-NPC: human neural progenitor cell; MBP: myelin basic protein; NGF: nerve growth factor; PDGF: platelet-derived growth factor; PLGA: poly(lactic-co-glycolic acid); PLL: poly-L-lysine; rhAm: recombinant human amelogenin.

*In situ* gelation without the need of light irradiation can be achieved using tyrosinase to induce enzymatic crosslinking, forming an injectable double network of HA-Tyr and gelatin [243]. This approach eliminates the need for invasive surgery and light application to crosslink the hydrogel. Enhanced cartilage regeneration in a rat osteoarthritis model was observed with encapsulation of Epigallocatechin-3-gallate (EGCG) in the HA-Tyr and gelatin hydrogel.

To overcome downsides associated with bulk hydrogel injection, such as shear damage to encapsulated cells and poor diffusion through the crosslinked implant, a microfluidic device was used to create an injectable suspension of micro tissues [244]. The micro tissues act as individual, high surface-to-volume environments that protect encapsulated cells during the injection process, and allow for great nutrient diffusion. Microgels of vinyl sulphonated HyA and Gel-SH were loaded with bone mesenchymal stem cells (BMSCs) and gelation was achieved through thiol-Michael addition. The microgels self-assembled into larger tissues through cell-cell interactions, were cultured for 1 week in chondrogenic medium, and then injected subcutaneously in nude mice. After 4 and 8 weeks of culture, the microgels demonstrated significantly greater tissue formation and lower CD31 and Collagen X expression compared to a suspension of BMSCs (Table 2). A similar study incorporated BMSCs in a collagen-tyramine precursor with HRP, crosslinked with HA-Tyr and H_2_O_2_ [245]. TGF-β1 was added to the cell-laden hydrogel group, which demonstrated improved cartilage regeneration in a preclinical model compared to empty defects and hydrogel-only treatments.

An alternative approach using subchondral bone as a fixation site permits the implantation of solid constructs, expanding viable materials beyond injectable HyA derivates. As cartilage and the subchondral bone possess different properties, bilayered compositions can be used [246]. This bilayered environment was recapitulated using a β-tricalcium phosphate (β-TCP) and hydroxyapatite (HyAp) base layer, covered with a hybrid gelatin-methacrylate (GelMA)-HyA hydrogel to mimic cartilage. hADSCs were encapsulated in the cartilage layer in either a 1:1 photocrosslinked GelMA-MeHA, or pure MeHA hydrogel. To further enhance regeneration in the chondral layer, acrylate-functionalised nano-silica (AFnSi) particles were incorporated. At both 8- and 12-weeks post-implantation in a rabbit model, the group containing hADSCs and AFnSi exhibited increased GAG deposition and defect coverage in the cartilage layer compared to other groups and untreated defects.

MeHA hydrogels can also serve as depots for the controlled release profile of biomolecules [247]. EVs from nutrient-deprived BMSCs and a vascular inhibitor were used to prevent expression of unwanted hypertrophy in a MeHA chondral layer and a thrombin-infiltrated methacrylate gelatin-CaCl_2_ bottom layer. The Gel-Ca layer was further divided into groups with or without thrombin, which is used to quickly coagulate infiltrating blood. Analysis in a rabbit osteochondral defect model after 8 weeks demonstrated enhanced collagen II deposition in the defect cartilage layer for the group containing all components as opposed to empty control, thrombin-free, and non-deprived EV groups.

Advances in HyA-based hydrogel development allows for the control of a multitude of properties, including stiffness, degradation, and stress-relaxation [248]. This degree of control, combined with its versatility of functionalisation, enables not only its combination with other materials, but the encapsulation of relevant biomolecules, and permits the development of well-defined materials, with different mechanical properties and biochemical cues [249]. As such, HyA-based hydrogels can be used as a foundation to develop materials to restore the zonal architecture of native cartilage, as well as guide zonal phenotypical differentiation of encapsulated or migrating chondrocytes.

#### 5.2.2 Bone

A co-polymer of MeHA and arginine-based unsaturated poly (ester amide) was shown to promote bone regeneration in preclinical calvarial defects [250]. Similarly, a composite 3D-printed nano HyAp-polycaprolactone (PCL) structure reinforcing a MeHA hydrogel encapsulating umbilical MSC-derived exosomes displayed significantly enhanced bone regeneration in preclinical calvarial defects after 8 weeks of implantation compared to control and exosome-free architectures (Table 2) [251]. The presence of the implant also induced enhanced expression of osteogenic markers including BMP-2 and VEGF-A (Table 2). As a delivery system, MeHA hydrogels demonstrated their capacity to steadily release encapsulated exosomes over a period greater than 20 days. The promotion of bone regeneration in this study by exosomes was hypothesized to be driven by high levels of microRNA 21, which is pro-angiogenic.

Conjugation of pyrogallol, an organic compound, to HyA hydrogels using EDC-NHS chemistry, can increase adhesive properties, enhancing fixation, one of the challenges of regenerating tissue *in situ* [252]. Pyrogallol-conjugated HyA can then be crosslinked through oxidation reactions with calcium phosphates additives such as whitlockite or HyAp. These bone compatible minerals also act as reinforcement to increase elastic modulus, while addition of BMP-2 significantly increases the hydrogels regenerative capacity of the material.

A nanocomposite hydrogel was developed using covalently crosslinked HA-Tyr, and a supramolecular assembly of HA-Tyr, peptide amphiphiles (GHK-Cu^2+^), and laponite [253]. This material demonstrated comparable bone regeneration to the commercially available Bio-Oss in a preclinical model of maxillary sinus floor reconstruction (Table 2).

As observed in the studies discussed, functionalisation of HyA-based hydrogels enables their use in bone regeneration as a versatile material for encapsulation, adhesion, and implantation. These possibilities make it a very attractive component of complex bone regeneration profiles to enhance not only bone regeneration by itself, but also to increase functionality of resulting materials.

#### 5.2.3 Muscle

HA-SH hydrogels have been functionalised with laminin-111, a key ECM protein involved in the embryological development of skeletal muscle [254]. Laminin-111 has been shown to support satellite cell migration and proliferation, key processes in muscle regeneration. When co-delivered with minced muscle grafts, laminin-111-functionalised HA-SH hydrogels demonstrated a restoration of neuromuscular function in a volumetric muscle loss (VML) model. Specifically, this combination resulted in a 42 % improvement in peak tetanic torque compared to untreated limbs, though the strength gains were not significantly greater than the minced graft control. Histological analysis revealed that while the HA-SH-laminin system did not substantially enhance the regenerative response, it reduced the macrophage activity and restored satellite cell content in the remaining muscle tissue after 14 days (Table 2). This emphasises the importance of further tuning HyA systems to fully support muscle repair.

Another approach developed MMP-cleavable peptide-crosslinked AcHyA hydrogels modified with RGD peptides and heparin to support cell adhesion and growth factor binding [255,256]. These hydrogels have demonstrated promise in preclinical models of both tibialis anterior (TA) and craniofacial VML injury. In the TA injury model, AcHyA hydrogels promoted enhanced functional recovery, muscle fibre growth, and native-like vascularisation, suggesting their ability to facilitate muscle regeneration and tissue repair (Table 2). Similarly, in a masseter injury model, AcHyA hydrogels improved muscle fibre cross-sectional area (FCSA), reduced fibrosis, and mitigated wound contraction at 8 and 16 weeks (Table 2). These findings in two different preclinical models highlight the potential of these AcHyA hydrogels to support muscle repair.

To address inflammation and tissue organisation, a biomimetic, layer-by-layer construct encapsulating PCL-melatonin (PCL-MLT) electrospun membranes into collagen I-functionalised HA-SH hydrogels was developed [257]. Collagen I was incorporated to support the formation of organised muscle fibres, a key aspect of effective muscle repair. PCL-MLT provided structural support while modulating the inflammatory response, reinforcing HyA's intrinsic anti-inflammatory properties to reduce fibrosis and promote tissue regeneration. In a VML model, this HA-SH/collagen I hydrogel system promoted muscle fibre alignment and organisation, improved tissue architecture, and reduced macrophage infiltration, contributing to a more efficient muscle repair process (Table 2).

Emerging solutions also integrate bioactive and conductive elements into HyA hydrogels. For example, hydrogels incorporating aminated HyA (AHA) and polyaniline (PANI) exhibit electrical conductivity to enhances myogenesis. In a recent study, self-healing conductive hydrogels based on AHA-PANI and oxidized hyaluronic acid were developed to engineer skeletal muscle [258]. These hydrogels demonstrated excellent cytocompatibility and promoted myogenic differentiation of C2C12 cells. In a preclinical TA VML model, the hydrogels demonstrated controlled degradation, reduced inflammation, and an upregulation in myogenic differentiation-related genes (Table 2). This suggests their potential to not only support skeletal muscle regeneration, but also modulate inflammation and facilitate tissue-specific healing. These advanced formulations, which integrate electrical conductivity with ECM-mimicking properties, represent a step forward for HyA-based hydrogels. By incorporating conductive elements and promoting cellular recruitment, these materials support myogenesis and enhance the regenerative processes in muscle repair.

In addition to functionalising HyA hydrogels with ECM components, bioactive molecules, or conductive elements, other approaches have focused on delivering a diverse array of myogenic progenitors directly to the injury site. As such, a photocrosslinkable HyA hydrogel, synthesised from an ester of HyA and a HyA photoinitiator, was developed for the localised delivery of satellite cells at the defect site [259]. This method not only supported muscle fibre repair and maintenance, but also promoted *de novo* muscle tissue formation. Alongside these regenerative effects, the HyA-based hydrogel facilitated the development of neural and vascular networks, contributing to overall tissue regeneration. Compared to control groups, these HyA-based hydrogels demonstrated enhanced tissue structure and functional recovery, highlighting their promising role in muscle repair applications (Table 2).

#### 5.2.4 Skin

In wound healing, HyA-based hydrogels are commonly modified to enhance functions like cell adhesion, inflammation control, and angiogenesis [240].

MeHA and MAHA photocrosslinked hydrogels have demonstrated effectiveness in supporting wound healing *in vitro* and *in vivo* [107]. *In vitro*, MeHA hydrogels were particularly effective in promoting the proliferation of human dermal fibroblasts (HDFs), human amnion-derived (HAD)-MSCs, and HUVECs. This result was attributed to the lower mechanical properties of MAHA compared to MeHA. *In vivo*, both MeHA and MAHA hydrogels supported complete wound healing within 14 days in a preclinical wound model. However, MAHA exhibited a more immediate and pronounced angiogenic response, peaking on days 1 and 3, while MAHA demonstrated higher initial CD11b + inflammatory cell counts, though these levels subsided by day 7, and no significant difference was observed by day 14 (Table 2).

Building on these foundational properties, advanced formulations incorporate bioactive compounds and tailored release profiles. For example, MeHA hydrogels were functionalised with the anti-inflammatory resveratrol and pro-angiogenic VEGF to treat burns [260]. This formulation resulted in controlled, sustained release of these factors, successfully reducing inflammation while promoting vascularisation, thereby accelerating wound closure over a 21-day period in a burn-induced splinted excisional wound model (Table 2). Such formulations emphasise the ability of HyA-based systems to integrate multiple pro-regenerative signals that are essential to manage chronic inflammation and stimulate angiogenesis.

HyA's versatility also enables its functionalisation with other biomaterials to support cell growth and structural integrity. The incorporation of GelMA with MeHA, for instance, has been shown to improve cellular network formation and the viability of encapsulated ADSCs over 21 days *in vitro*, as well as increase vascularisation by over threefold in a chick chorioallantoic membrane (CAM) assay compared to MeHA only [261]. This adaptability of HyA-based hydrogels to support cellular integration and vascularisation make it well-suited to create a pro-healing environment for full-thickness wounds (Table 2).

HyA-based nanocomposite hydrogels, such as those combining HA-CHO, hydrazide-modified HyA, and aldehyde-modified cellulose nanocrystals, illustrate the benefits of HyA in wound repair by providing a robust matrix that can adapt to tissue dynamics while sustaining cell-instructive cues [83]. In this system, HyA's modifiable structure allows the formation of acylhydrazone bonds, creating a mechanically strong and self-healing hydrogel suitable for dynamic wound environments. Furthermore, HyA's ability to form covalent bonds with platelet-rich plasma (PRP) components enables controlled release of growth factors, enhancing granulation tissue formation, collagen deposition, re-epithelialisation, and neovascularisation in preclinical models of full-thickness skin wounds (Table 2).

A promising approach to treat diabetic wounds involves the use of pH-responsive HyA hydrogels, which are particularly valuable in wounds where the local pH fluctuates, enabling the targeted release of therapeutic agents and accelerated healing by aligning with the needs of chronic wounds [262]. The combination of HA-CHO and HA-ADH with collagen has been shown to accelerate degradation at a slightly acidic pH of 6.5 compared with pH 7.4 [263]. This increased degradation rate enabled the rapid release of metformin-loaded particles at higher pH, which led to enhanced healing by reducing inflammation, promoting collagen deposition, and supporting neovascularisation in a diabetic wound model after 12 days (Table 2).

Other approaches leverage HyA's intrinsic properties for wound-specific adaptations. For instance, γ-PGA-SH and HA-CHO, formed through a rapid thiol-aldehyde reaction, create self-healing networks that mimic the viscoelasticity of the ECM and possess antioxidant properties. These HyA-based hydrogels enhanced wound healing by promoting angiogenesis and collagen deposition in full-thickness models (Table 2) [79].

A promising strategy to accelerate tissue repair involves targeting the migration of key cell types, including fibroblasts and endothelial cells. To enhance this process, a HA-Tyr-chitosan hydrogel was developed incorporating copper-doped bioglass (ACuBG) nanoparticles, which provide a sustained release of silicon and copper ions to induce migration of endothelial cells and neovascularisation [264]. This multi-crosslinked HyA hydrogel exhibited improved mechanical properties compared to HA-Tyr or chitosan-only hydrogels. In preclinical models of full-thickness skin defects, the HyA hydrogel supported complete wound closure with the presence of hair follicles and sebaceous glands within two weeks (Table 2).

#### 5.2.5 Neural tissue

HyA-based hydrogels have also been developed to enhance the repair of neural tissue. For example, the incorporation of ECM components, such as RGD-based peptides, enhances neural repair by improving cell adhesion, migration, and differentiation. Peptide-decorated AcHyA systems crosslinked with an MMP-cleavable peptide formed microporous hydrogels that mimic the brain's native microenvironment. These hydrogels facilitated integrin-mediated interactions and rapid cellular infiltration. When injected into the stroke cavity, these microporous hydrogels supported astrocyte infiltration, reduced inflammation and scarring, and enabled NPC migration up to 300 μm into the hydrogel, guided by co-localised astrocytes. This demonstrates the potential of ECM-integrated HyA hydrogels in promoting endogenous regeneration after stroke [265] (Table 2).

Further enhancing stem cell survival and differentiation, AcHyA hydrogels have been functionalised with bone morphogenic protein-4 (BMP-4) and brain-derived neurotrophic factor (BDNF), coupled with heparin to sequester these growth factors effectively. These functionalised hydrogels replicate critical ECM characteristics by providing both structural integrity and localised biochemical signals necessary for neural differentiation. Crosslinking with MMP-sensitive peptides ensures enzymatic degradation in response to local remodelling, facilitating human neural progenitor cell (iPS-NPC) migration and integration. *In vivo*, these hydrogels demonstrated significant improvements in encapsulated iPS-NPC survival, neuronal differentiation, and angiogenesis within two weeks of implantation, highlighting their therapeutic potential for stroke repair [266] (Table 2).

Injectable HA-SH hydrogels functionalised with gelatin and crosslinked with PEG diacrylate (PEGDA) were used to optimise mechanical properties and gelation times, addressing challenges in stem-cell based CNS therapies such as poor cell survival and integration. Soft hydrogels (elastic modulus <10 Pa) promoted neural stem cell (NSC) network formation and neuronal differentiation. In comparison, stiffer hydrogels (elastic modulus 10 and 100 Pa) favoured spheroid growth and reduced proliferation rates, contributing to a regeneration-permissive microenvironment that enhances cell viability and differentiation potential [267]. Incorporating maleimide-functionalised PCL nanofibres further stabilised the hydrogel's mechanical properties while significantly improving spinal cord tissue regeneration in a contusion injury model [268]. Over 28 days, the hydrogel composite reduced spinal cord thinning, increased cell infiltration, and promoted axonal growth, neurogenesis, and pro-regenerative M2 macrophage polarization, as evidenced by higher axon density and β-III-tubulin (Tuj1)-positive cells (Table 2).

In addition, HyA-based hydrogels can direct neural differentiation by tuning mechanical properties, such as the degree of substitution in MeHA hydrogels. Hydrogels with stiffness similar to neonatal brain tissue promoted neuronal differentiation, with cells exhibiting long, branched processes and mature neuronal marker β-III tubulin expression after three weeks. In contrast, stiffer hydrogels resembling spinal cord tissue supported astrocyte differentiation, essential for neuroprotection and repair [269]. *In vivo*, high MW HyA reduced astrocyte proliferation and chondroitin sulphate proteoglycan (CSPG) expression 10 days post-injury in a rat model of spinal dorsal hemisection injury (Table 2) [270].

Electroconductive HyA hydrogels represent a promising avenue for advancing neural tissue engineering and broader neural repair strategies. For instance, catechol-functionalised HyA (HA–CA) hydrogels incorporating carbon nanotubes or polypyrrole exhibit improved mechanical properties, slower degradation, and high electroconductivity, enabling electrical signal transmission, critical for NSC differentiation and maturation [271]. These preclinical findings underscore the potential of electroconductive hydrogels to replicate the electrical properties of neural tissue and enhance regeneration. Beyond applications in the CNS, electroconductive HyA hydrogels have also shown promise in peripheral nerve regeneration, where conductive properties play a pivotal role in axonal repair and functional recovery. For example, HyA-based hydrogels **functionalised with cystamine and polypyrrole** provided a supportive microenvironment for nerve repair by promoting **Schwann cell activity** and **axonal growth** [272]. These self-healing and conductive hydrogels activated key signalling pathways such as **IL-17** and **mitogen-activated protein kinase (MAPK),** which are involved in **myelination** and **axon guidance.** In **rat models** of peripheral nerve injury, these hydrogels supported functional recovery, including improved axonal growth and reduced scar tissue formation (Table 2).

## 6 Discussion

The diverse range of properties, modularity, and potential for functionalisation of HyA highlighted in this review underscore its significance in biomedical research as a versatile material for disease modelling and therapeutic interventions. HyA hydrogels have demonstrated significant promise in tissue repair and regeneration in multiple, diverse preclinical models. Applications include osteochondral repair, where functionalised HyA hydrogels promote osteogenesis and chondrogenesis, and wound healing, where HyA hydrogels with anti-inflammatory and vasculogenic modifications improve wound closure. Additionally, the ability of HyA-based hydrogels to support vascularisation further enhances their potential in large-scale tissue reconstruction.

HyA hydrogels provide versatile platforms for modelling of disease mechanisms in 3D and supporting high-throughput drug screening, by providing a highly modular ECM in these systems. This ability to mimic the ECM is critical, as it provides a biologically relevant microenvironment. For instance, appropriately-designed HyA hydrogels enable the study of tumour microenvironments, fibrosis, and immune cell interactions, facilitating the development of more targeted therapies. Additionally, HyA-based hydrogels are particularly valuable for organ-on-a-chip models, which frequently lack a suitable ECM to accurately represent tissue physiology [59]. By incorporating HyA hydrogels with suitable functionality and signalling, these models can more faithfully recapitulate the natural ECM, enhancing their relevance for drug screening and disease modelling. Beyond cancer, HyA-based systems have supported research into inflammatory diseases, enhancing our understanding of complex pathological processes and enabling screening for therapeutic candidates [224].

Innovative strategies like one-pot reactions and click-chemistry-based approaches, are being developed to reduce synthesis time, improve reproducibility, and minimise the need for extensive purification, enhancing the feasibility of large-scale production [69,281]. For instance, copper-free click chemistry enables rapid and biocompatible modifications without introducing cytotoxic reagents, making it particularly promising for clinical translation [282].

## 7 Shortcomings

Despite these advantages and impressive developments in the field, few HyA-based hydrogels have emerged as therapeutic interventions. Unmodified HyA is widely used clinically due to its inherent biocompatibility and biodegradability, but its functionality and half-life are limited, requiring repeated dosing. Chemical modifications of HyA aim to enhance its utility; however, balancing safety, functionality, and translational feasibility is crucial to identify the most promising candidates for clinical use.

Α key consideration in achieving clinical translation is simplicity in synthetic ECM design as overly complex designs may hinder reproducibility, scalability, and regulatory approval [283]. Instead, focusing on core design parameters such as ligand and growth factor presentation, enzymatic degradation, material mechanics, and matrix architecture ensures that synthetic ECMs effectively coordinate intrinsic and extrinsic determinants of cell fate to promote successful regeneration. Importantly, these design considerations must be tailored to the intended application, as the balance between complexity, functionality, and simplicity depends on the specific disease or therapeutic context.

Current functionalisation methods for HyA often rely on the physical incorporation of bioactive molecules, limiting their potential. Advanced chemical crosslinking and site-specific conjugation allow HyA-based hydrogels to exhibit targeted and stimuli-responsive behaviours, supporting drug delivery and wound healing, and addressing therapeutic needs such as anti-inflammatory effects, tissue regeneration, and antimicrobial activity [112,113,284].

Additionally, identifying the appropriate MW of HyA for specific therapeutic needs is essential. High MW derivatives are particularly suited to reducing inflammation, making them attractive for applications in chronic inflammatory diseases and wound healing. On the other hand, low MW HyA derivatives are being explored for their distinct bioactivity, particularly their ability to transiently activate immune responses. This property may be beneficial in contexts like early wound healing or vaccine development. These contrasting effects highlight the importance of MW selection and customisation when designing HyA-based hydrogels.

## 8 Future directions

Moving forward, future developments in HyA-based hydrogels will focus on enhancing precision and scalability while incorporating a more diverse range of bioactive agents. For instance, current advances involve novel combinations of chemical modifications, such as the incorporation of phenylboronic acid and quaternary ammonium chitosan within the same system, enabling pH-responsive and reversible interactions alongside antimicrobial activity and enhanced cell adhesion [285].

Modulation of the immune response is another critical area where HyA hydrogels hold promise. New immunomodulatory targets and molecules are constantly being identified, including immune metabolites, which present novel opportunities for targeted treatment strategies. In this context, HyA hydrogels can serve as powerful platforms and enabling technologies for the effective delivery of compounds such as succinate or itaconate [[286], [287], [288]]. Recent studies have revealed that these metabolites have anti-inflammatory effects on macrophages, but also have an innate ability to inhibit ROS production as well as possessing antiviral properties, making them promising therapeutic candidates acting on multiple pathways [[289], [290], [291]].

Another particularly promising application of these advancements lies in regenerative medicine. HyA hydrogels are increasingly used as bioinks for 3D bioprinting, where techniques like microprinting and photocrosslinking provide spatiotemporal control over the positioning and release of bioactive agents. These methods enable the creation of complex 3D tissue structures with precise geometries and controlled biochemical environments. Furthermore, bioink printing can be combined with conventional 3D printing or advanced methods such as electrowriting to create composite constructs with enhanced mechanical and structural properties, broadening their potential applications [292,293]. For example, 3D-printed HyA hydrogels have shown promise in vascularised tissue constructs, which are essential to support the viability of large-scale tissue implants. Future research should focus on optimising these techniques for clinical scalability while maintaining reproducibility and safety.

Emerging areas of development include cell-based crosslinking strategies, where cells themselves participate as building blocks in hydrogel formation, enabling the development of living HyA hydrogels capable of adapting to their environment and integrating more effectively with host tissues [90]. Similarly, supramolecular assemblies of HyA, offering tuneable mechanical properties including stress-relaxation, are actively being explored for tissue repair as they offer the potential for injectable and sustained therapeutic delivery [[294], [295], [296]]. Both approaches represent transformative advancements in the development of more tissue-mimetic, “living” HyA-based hydrogels, capable of dynamic interactions with their microenvironment.

## 9 Outlook

The next phase of development of HyA hydrogels should prioritise the integration of advanced, multi-functionalisation techniques with novel fabrication strategies. This includes leveraging cell-based crosslinking and supramolecular assemblies to create dynamic, "living" hydrogels that can respond to environmental changes and support tissue integration. Additionally, there is a need to focus on optimising these approaches for clinical scalability while maintaining safety and reproducibility.

Ultimately, these innovations aim to drive HyA-based hydrogels toward clinical application, expanding their use in regenerative medicine, drug delivery, and disease modelling, fostering new therapeutic opportunities.

## CRediT authorship contribution statement

**Noémie Petit:** Writing – review & editing, Writing – original draft, Conceptualization. **Yu-yin Joanne Chang:** Writing – review & editing, Writing – original draft. **Franz Acker Lobianco:** Writing – review & editing, Writing – original draft. **Tom Hodgkinson:** Writing – review & editing, Funding acquisition. **Shane Browne:** Writing – review & editing, Supervision, Funding acquisition, Conceptualization.

## Funding sources

The authors acknowledge support from Research Ireland under Grant Number 13/RC/2073_P2, and co-funded by the European Union (EU) under MedTrain+Grant Agreement No. 101081457; LifETIME-CDT Grant No. 8/EPSRC-CDT/3583; The European Federation for the Study of Diabetes/Lily European Diabetes Research Programme; the Health Research Board Grant No. EIA-2022-009; and Research Ireland Pathway Award No. 21/PATH-S/9306.

## Declaration of competing interest

The authors declare that they have no known competing financial interests or personal relationships that could have appeared to influence the work reported in this paper.

## Data Availability

No data was used for the research described in the article.
